# Exploring the associations between prenatal PCB exposures and gene expression: Observations from a study of newborn Slovak infants

**DOI:** 10.1016/j.ecoenv.2025.119059

**Published:** 2025-09-20

**Authors:** Amara Saleem, Andrew Volz, Tanmoy Mondal, Christopher A. Loffredo, Tomas Trnovec, Lubica Palkovicova Murinova, Kamil Conka, Beata Drobna, Somiranjan Ghosh

**Affiliations:** a Department of Oncology, Georgetown University, Washington, DC 20057, USA; b Departments of Biology, Howard University, Washington, DC 20059, USA; c Department of Environmental Medicine, Slovak Medical University, Bratislava, Slovak Republic; d Department of Toxic Organic Pollutants, Faculty of Medicine, Slovak Medical University, Bratislava, Slovak Republic; e Departments of Pediatrics and Child Health, College of Medicine, Howard University, Washington, DC 20059, USA

**Keywords:** Polychlorinated Biphenyls (PCBs), Gene Expression, Exposures, New-Born Children, Slovakia

## Abstract

Polychlorinated biphenyls (PCBs) are persistent organic pollutants known to have deleterious effects on child and adult development; however, less is known about the relationship between mother-newborn exposure levels. The objective of this study is to understand prenatal PCB exposure and its association with gene expression, using data from a cohort of exposed mothers and infants in eastern Slovakia. For 91 mothers and infants participating in this study, serum PCB concentrations were determined using gas chromatography and high-resolution mass spectrometry. Affymetrix microarray was performed utilizing Human Genome U133 Plus 2.0 gene chip. Statistical analysis compared the genetic expression levels of high versus low exposure mother-infant pairs (< 368.57 ng/g lipid vs. > 368.57 ng/g lipid). Statistical analysis results showed that mother’s blood and cord blood PCB concentrations were highly correlated. Results showed that higher levels of PCB concentrations were associated with differential expressions of multiple genes. Global gene analysis identified significant dysregulation of genes XIST, EXOC6B, EIF1AY, and RPS4Y1. Gender-based gene analysis identified significant dysregulation of genes RUFY1, S100A8, ELOVL7, FXYD3, DEFB124, and DAB2. Further such investigations should be implemented to confirm these observations and more fully define the legacy of environmental PCB contamination.

## 1. Introduction

### 1.1. PCB exposures and their health effects in Slovakia

Polychlorinated biphenyls (PCBs) are persistent organic pollutants that were inappropriately dumped into the environment as a legacy of chemical manufacturing in many areas of the world. In eastern Slovakia, unlawful dumping of PCBs over several decades caused extensive pollution of freshwater sediments (Kocan et al., 2001; Park et al., 2007; Wimmerová et al., 2015). Despite the banning of such practices in Slovakia in 1984, studies between 1987 and 1990 reported high concentrations of PCBs in locally grown food (Hertzman, 1995). A study of breastfeeding mothers in the highly polluted Michalovce district showed that concentrations of PCBs in breast milk averaged from 4.0 to 4.4 mg/kg lipids (Hertzman, 1995), which significantly exceeds regulatory safety levels (< 0.01–0.04 ng/g) (Korrick and Altshul, 1998). Assessments of the newborn population of this region have shown deleterious effects in neuro-behavioral development and reduction in thymus size at birth (Park et al., 2008; Šovčíková et al., 2015). Additional studies have reported associations of PCB exposures with chronic diseases and disorders, including reproductive health effects (Plíšková et al., 2005), neurological deficits (Park et al., 2009), immune system alterations (Jusko et al., 2016), endocrine effects (Rádiková et al., 2008), and hearing loss (Trnovec et al., 2008), while gene expression studies have reported disruptions in key mechanistic pathways involving diabetes, cardiovascular diseases, and cancers (Ghosh et al., 2014; 2015, 2018). Developmental effects from PCB exposure have also been reported (Royland et al., 2008).

### 1.2. Prior research on PCBs by our team

Gene expression studies by our group on persons environmentally exposed to PCBs in Slovakia (Ghosh et al., 2015; 2018) have reported associations of PCB exposures with alterations in the expression levels of multiple genes in disease and disorder pathways that are in accord with earlier studies (Mitra et al., 2012; Ghosh et al., 2013; 2014). Comparing and contrasting the gene expression levels in higher and lower exposure regions led to the identification of numerous genes that were markedly under- or over-expressed in relation to the levels of PCBs measured in the blood of these research subjects. Recently, our gene expression studies of children in Slovakia revealed that early-life exposures to PCBs and other organochlorine compounds were associated with significant alterations across several pathogenetic pathways. These included downregulation of genes such as LEPR, CYP2D6, and MYC, which are involved in metabolic, endocrine, and xenobiotic processes, with some variation by sex (e.g., CYP2D6, LEPR) and ethnicity (Mondal et al., 2022). These prior studies identified downregulation in key metabolic, endocrine, and xenobiotic pathway genes, with some differences by sex and ethnicity (Mondal et al., 2022).

### 1.3. Knowledge gaps on the effects of PCBs in infancy

Given the breadth of knowledge generated in the studies listed above, there are numerous health concerns about the long-term legacy of PCB environmental pollution in Slovakia. Yet important gaps persist, particularly on the relationship of maternal and newborn exposure levels, and their potential effects in children’s health. Previously we examined PCB-associated transcriptional alterations in older children from this cohort, identifying changes in metabolic, endocrine, and xenobiotic pathway genes using targeted approaches (Ghosh et al., 2015; Mondal et al., 2022). However, these earlier studies did not evaluate molecular changes at birth, nor did they analyze paired maternal and cord blood samples or perform genome-wide expression profiling. In this study, we obtained stored blood specimens from mother-infant pairs collected at the time of delivery, together with prior measurements of a wide range of PCB congeners in these same specimens. Our aims were to investigate the relationship between mother and infant levels of PCBs, conduct gene expression measurements, and relate exposure and gene expression to maternal demographic factors and newborn sex and birthweight.

## 2. Materials and methods

### 2.1. Study population

During 2007–2008, mothers with newborns (N = 220) from the Michalovce region (highly contaminated by PCBs) were recruited for the project “Environmental Exposure to PCB and Development of Nervous System in Children.” Recruitment was based on the following inclusion criteria: pregnancy and delivery without significant morbidity, full-term pregnancy (37–42 weeks), and the mother’s 1st or 2nd pregnancy. At delivery, samples of maternal and cord blood were collected and processed, and blood serum was stored for analysis. Children were followed to the age of 10–12 months for the project “Effects of PCB and dioxin exposure on mental and psychomotor development of infants” when fresh samples of blood serum were collected and stored frozen for further PCB analyses. For a subgroup of this total cohort, we selected 91 mother-infant pairs (out of 180 total pairs) for a gene expression study that is reported in this manuscript.

### 2.2. Biospecimen collection, processing and storage

Rare paired maternal–cord blood samples (20 mL each) from a high-exposure region were collected during infant delivery. Whole blood was collected in PAXgene^®^ Blood RNA Tubes (IVD, BD, Cat. # 762165), stored in −80 °C, and subsequently shipped to the Molecular Genetics Laboratory at Howard University, Washington, DC, on dry ice, according to the protocol of the manufacturer (Qiagen, MD). Specimens were stored again in −80 °C prior to gene expression assays.

### 2.3. Analysis of PCB exposure in the samples of maternal and cord blood

Concentrations of PCBs in blood serum samples were measured at the Slovak Medical University in Bratislava, Faculty of Medicine, Department of Toxic Organic Pollutants, using high resolution gas chromatography/high resolution mass spectrometry (HRGC/HRMS). Details of analyses were published and have been previously reported (Conka et al., 2005; Jursa et al., 2006). To maximize the contrasts in gene expression, we selected stored biospecimens from above and below the median value of the distribution of total PCB in the 91 pairs, and we also tried to balance the numbers of males and females, resulting in 29 samples (16 males, 13 females) in the gene expression subset.

### 2.4. Gene expression and pathway enrichment analysis

Global differential gene expression analysis was performed on whole blood-derived RNA from 29 selected children (boys: n = 16, girls: n = 13), following the protocol established in our previous work by Ghosh et al. (2011). Oligonucleotide microarray experiments were conducted by EpigenDx (Boston, MA) using the Affymetrix HG-U133 Plus 2.0 Array platform, which offers comprehensive coverage of the entire transcribed human genome on a single array (Mondal et al., 2023, 2024). Differentially expressed gene sets were analyzed using microarray results and a one-way ANOVA model via Transcriptome Analysis Console (TAC) software (Thermo Fisher Scientific, US). Probe summarization and probe set normalization were performed using the GC-RMA algorithm, which includes GC-RMA background correction (FDR controlled), quantile normalization, log2 transformation, and median polish probe set summarization (Wu and Irizarry, 2004). The study controlled for a false positive rate below 5 %, with a statistical significance threshold of 0.05.

From the differential gene expression datasets described above, the identification of cellular processes and pathways by IPA (Ingenuity^®^ Systems, http://www.ingenuity.com) was performed according to the method described in our earlier study (Mondal et al., 2023, 2024). Briefly, datasets comprising gene identifiers and corresponding expression values (differential expression with significant fold change at p < 0.05) from the microarray experiment were imported into IPA. Differentially expressed gene identifiers were mapped to related changes in biofunctions. The networks were generated algorithmically based on their connectivity. Using IPA, we identified the top network by incorporating a large set of differentially expressed genes with the goal of uncovering the most extensive array of relationships among the focus genes. Networks were “named” on the most prevalent functional group(s) present.

### 2.5. Statistical analysis

We began by examining demographic variables including the age of mother at delivery, whether the mother lived in the region her whole life, type of delivery, newborn birth weight, and newborn sex. Using chi-square tests for categorical variables and t-tests for continuous variables, we assessed the demographic similarities of gene-expression cohort (N = 91, with complete PCB and demographic data) and molecular study subgroup (N = 29, with complete PCB, demographic, and gene expression data). This (N = 29) subgroup represented a sample of those participants with the highest and lowest exposure levels of PCBs.

**Fig. 1.**
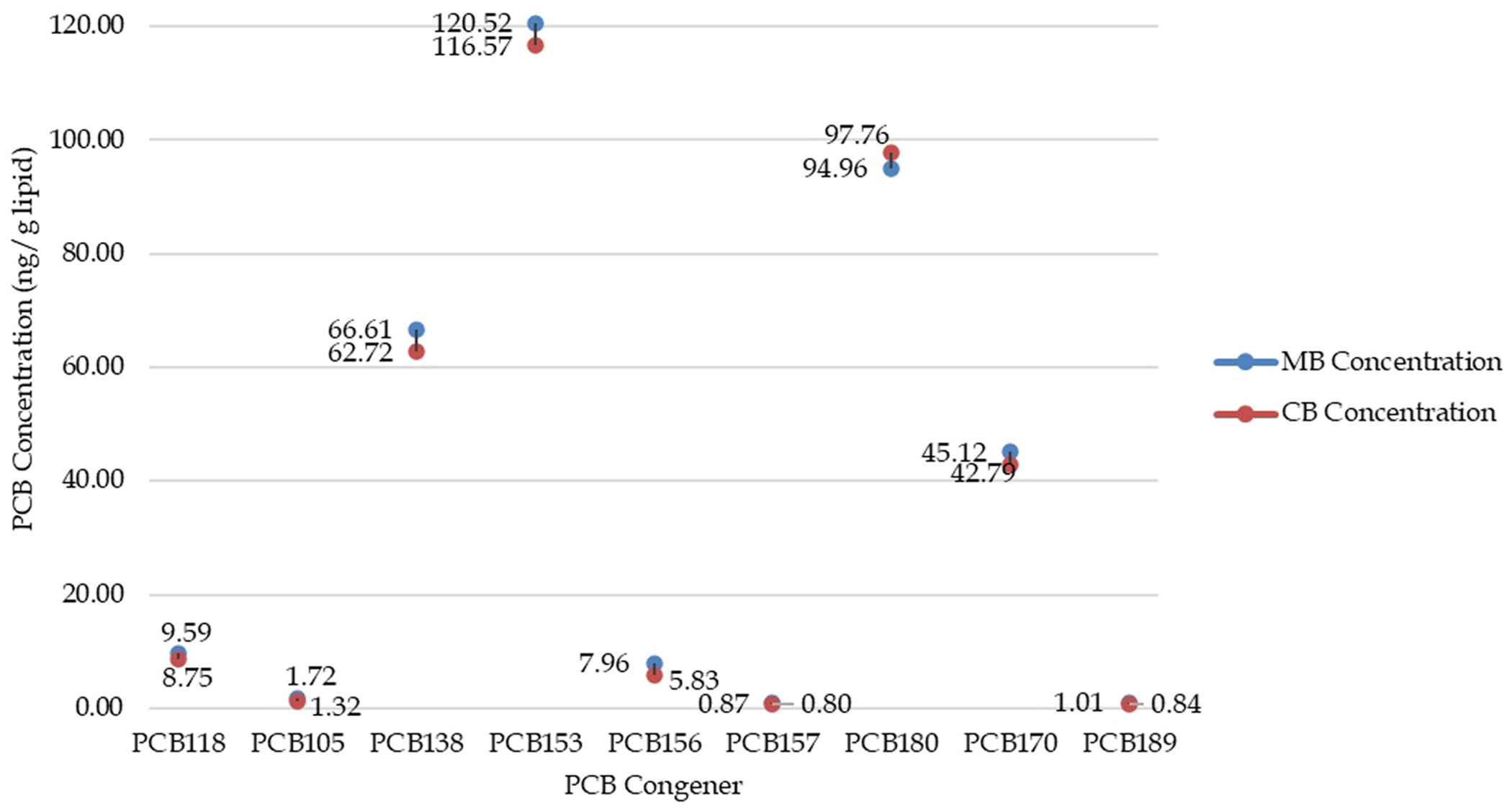
Mothers blood and cord blood concentrations for selected PCB congeners in molecular study subgroup: Mothers blood versus cord blood PCB concentrations. Scatterplot showing the relationship between the average PCB concentration (ng/g lipid) in mothers’ blood versus cord blood for participants in the molecular study subgroup.

We then divided the molecular study subgroup using the median total PCB exposure for mother’s blood (MB) and cord blood (CB). All mother-child pairs with exposures above the median were considered high PCB exposure, and all pairs below the median were considered low PCB exposure. We assessed the demographic characteristics of high versus low exposure individuals in the molecular study subgroup, using chi-square tests for categorical variables and t-tests for continuous variables. T-tests were performed to assess mother’s blood and cord blood PCB concentration associations between the gene-expression cohort and molecular study subgroup. To assess the association between maternal blood and cord blood PCB levels within the molecular study subgroup, a Spearman correlation analysis was performed. Log-transformed PCB concentrations were used to evaluate the strength and direction of relationships between the two sample types. The results are presented in Fig. 1.

Nine selected PCB congeners were analyzed because they represent 75 % of total PCB exposure (Sonneborn et.al., 2008). Congeners 118, 105, 156, 157, and 189 contributed less than 5 % each to the total PCB concentration for mother’s blood concentrations for the subgroup, while congeners 153, 138, 180, and 170 contributed between 11 % and 25 % each. The levels for these congeners were analyzed for mother’s blood versus cord blood for the molecular study subgroup. A nonparametric Wilcoxon Rank-Sum (Mann–Whitney U) test was performed to compare Total PCB levels between MB and CB, as well as gender-wise differences, gene expression levels, and differences among PCB types.

Focusing on genes that had a fold change greater than + /− 1.5, an analysis was conducted to study the relationships between such genes and total PCB levels in the molecular study subgroup. Linear regression models were used to determine changes in the gene expression per increase in total PCB (ng/g), generating P-values with and without adjustment for maternal age and the sex of the child. It was necessary to remove one outlier, i.e. who had an extremely high total PCB level that exerted profound influence on the analysis: results are reported with and without the outlier. Statistical Analysis Software (SAS version 9.4) was used for all analyses. A p-value < 0.05 was considered statistically significant.

## 3. Results

### 3.1. Cohort and exposure subgroups and analysis

From the gene-expression cohort containing 91 mother-child pairs, we selected 29 pairs for a molecular study subgroup, as described in detail above. The demographic statistics for the cohort and subgroup are shown in Table 1. There were no significant differences between the cohort and subgroup for any of the five demographic variables (p > 0.05 for all comparisons).

There were no significant differences between the gene-expression cohort and molecular study subgroup mother’s blood PCB concentrations (p > 0.05). There was a statistically significant difference between the gene-expression cohort and molecular study subgroup cord blood PCB concentrations (gene-expression cohort, median = 287.8, molecular study subgroup median = 368.6, p = 0.034) (Table 2).

Within the molecular study subgroup, we found that the high-exposure mothers were significantly older than the mothers in the low-exposure group (Supplemental Tables 13, 14). In mothers’ blood, the mean ages were 23.8 vs. 27.9, and in CB, the mean ages were 23.8 vs. 27.6, for high vs. low exposure groups respectively (p = 0.012 for MB, p = 0.022 for CB). Regarding birth weight of the newborn, type of delivery, sex of the newborn, and length of mother’s residence in the region, there were no significant differences between high-exposure pairs and low-exposure pairs for either mother’s blood or cord blood data set.

### 3.2. PCB exposures in mothers and newborns

We confirmed that the total PCB concentration in the mother’s blood was correlated with the concentration in cord blood. There was no statistically significant difference between the total mother’s blood and total cord blood PCB concentrations. The range of individual PCB concentrations, from the lowest to the highest, in cord blood and maternal blood was recorded to illustrate the distribution in the molecular study subgroup (Supplementary Figures 1 and 2). Fig. 1 compares the mothers vs. cord blood mean concentrations for nine different PCB congeners, which represent approximately 75% of total PCB exposure (Sonneborn et.al., 2008). Without the outlier, there was a statistically significant association between the congener PCB concentrations of the mothers vs. cord blood (r = 0.625, p < 0.0001). With the outlier included, there was still a statistically significant correlation (r = 0.894, p < 0.0001). The relationship between maternal age and total PCB levels in maternal and cord blood was assessed and showed no significant association with total PCB concentrations, although a positive trend was observed in the scatter plot (Supplementary Figure 12).

**Fig. 2.**
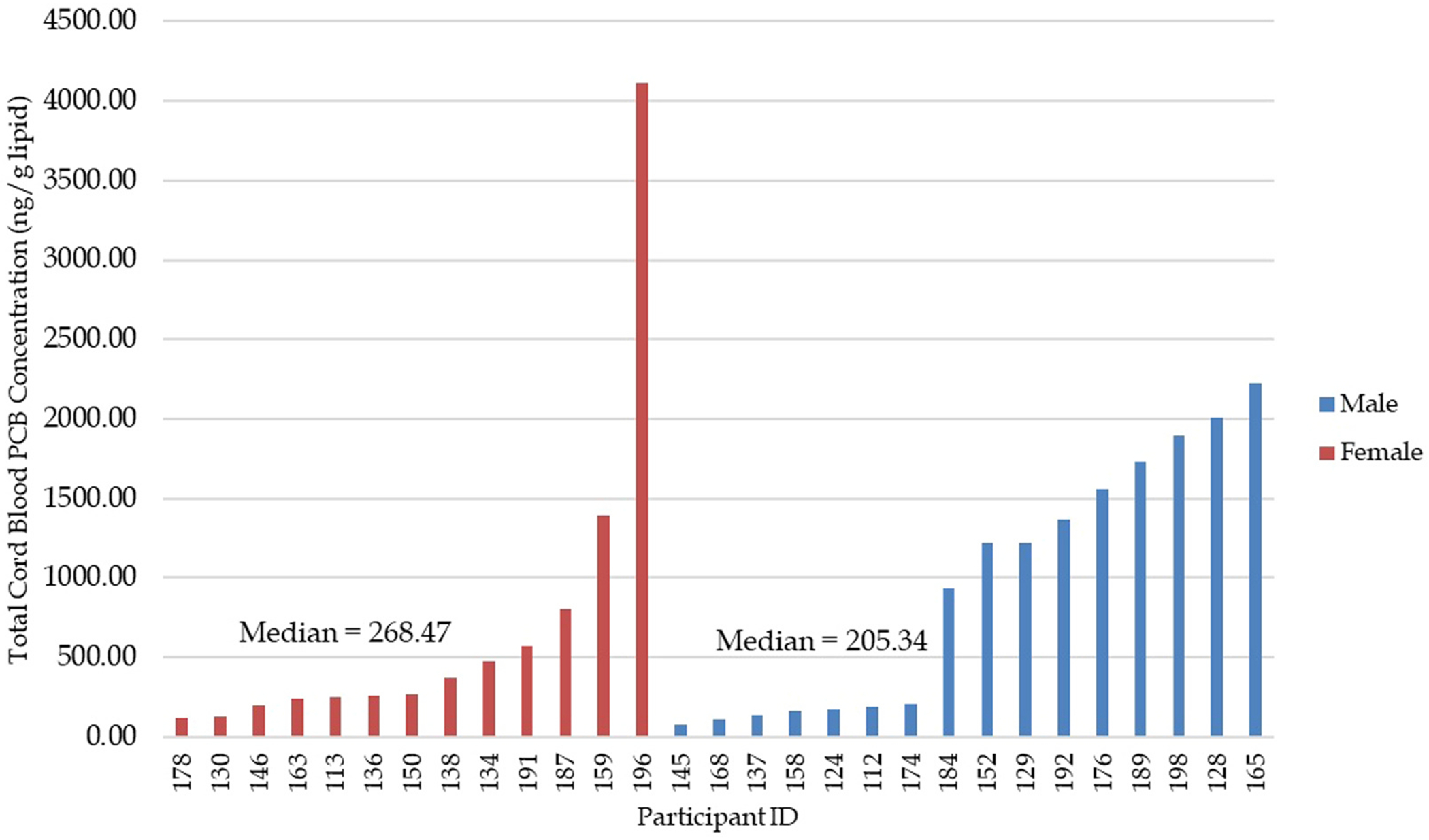
Individual participant cord blood concentrations of total PCB for the molecular study subgroup, arranged from lowest to highest exposure and separated by gender: Individual participant cord blood PCB concentration (ng/g lipid) arranged by gender and exposure. Bar chart showing all individuals in the molecular study subgroup, their total cord blood PCB concentration, and their gender.

Fig. 2 compares male vs. female total cord blood PCB concentrations. Without the outlier, there was not a statistically significant difference in the total cord blood PCB concentrations between male vs. female individuals (t= 0.703 p= 0.488). With the outlier included, there was a statistically significant difference (t= 2.14, p= 0.042). One participant had exceptionally high total PCB concentrations (maternal blood: 5921.70 ng/g lipid; cord blood: 4113.51 ng/g lipid), exceeding the next highest value by more than 6-fold for maternal and 4.9-fold for cord samples. Inclusion of this point substantially altered correlation coefficients and gene expression results due to leverage effects. To minimize distortion of the general exposure–response relationship, primary analyses were conducted without this outlier; however, exact outlier values are presented in the Supplementary Materials. Additionally, we reanalyzed total PCB levels in maternal blood and cord blood using the two-sided nonparametric Wilcoxon rank-sum (Mann–Whitney U) test, and found no significant association between total PCB levels and either maternal–cord blood pairs or gender differences (Supplementary Table 13). The Wilcoxon rank-sum (Mann–Whitney U) test for individual PCB types in maternal blood and cord blood showed that PCB202 exhibited a significant difference both with and without the inclusion of outliers (Supplementary Table 14).

### 3.3. Gene expression: global analysis

The global results for the genes that showed upregulation are shown in Supplementary Table 1, whereas genes that showed downregulation are shown in Supplementary Table 2. Within the upregulated genes, it was found that multiple genes had a statistically significant elevation in relation to the total PCB levels. When left unadjusted, 10 genes were found to be significantly over-expressed at average fold change < −1 or > 1 and p-value 0.05, in relation to the levels of total PCB (Table 3). After adjusting for maternal age and sex of the child, four total genes were found to be significantly upregulated in relation to the total PCB levels: *EXOC6B* (p= 0.034), *PAM* (p= 0.039), *CLU* (p= 0.037), and *XIST* (p= <0.001).

**Fig. 3.**
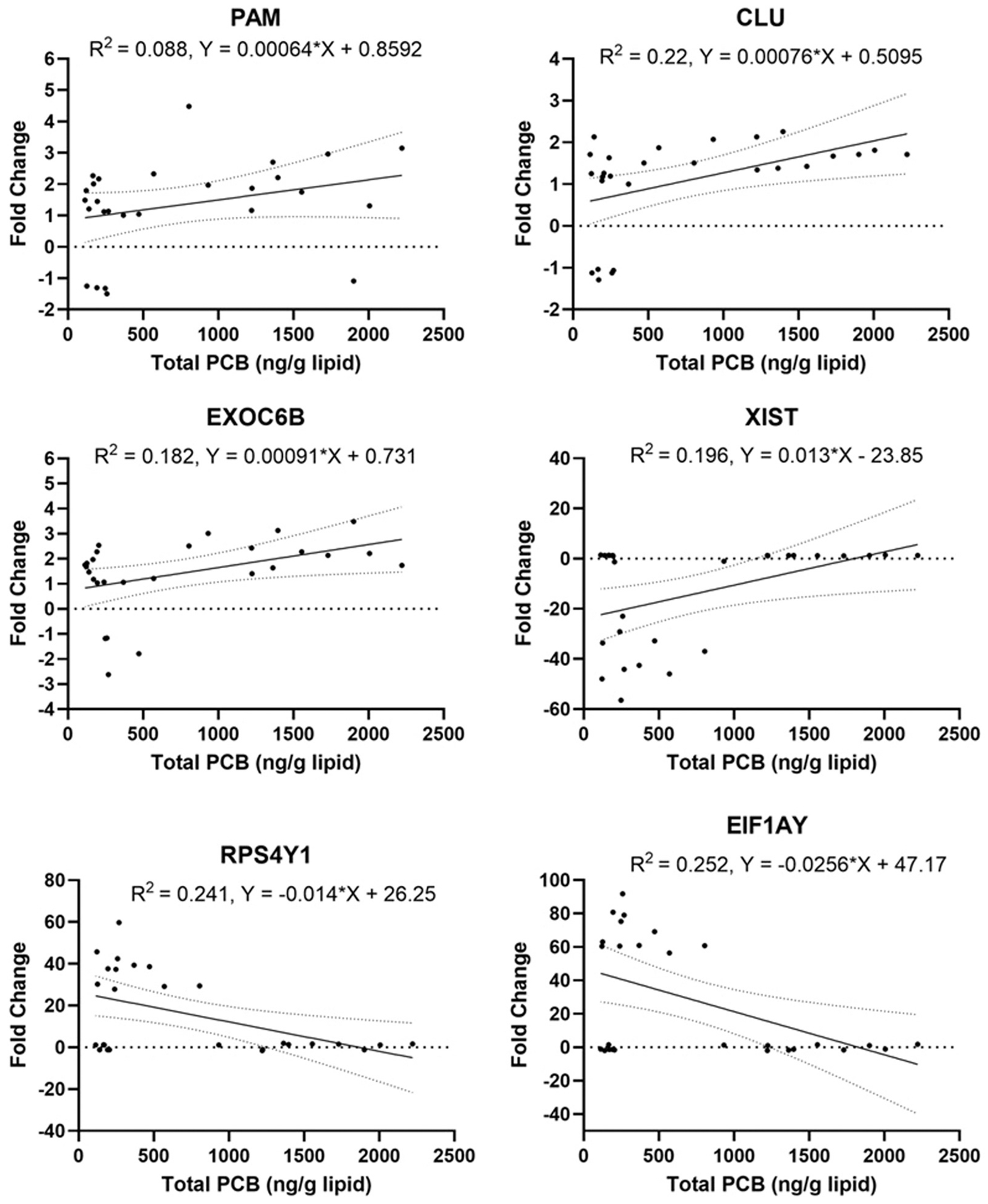
Total PCB concentration and differential expression for significantly altered genes: Total PCB (ng/g lipid) concentration versus expression of significantly altered genes. Linear regression plot analysis showing the relationship between total PCB concentrations and the differential expression (fold change) of significantly altered genes (PAM, CLU, EXOC6B, XIST, RPS4Y1, and EIF1AY) across individual participant samples. Outlier samples were not included in the current plot. *Statistically significant at p < 0.05.

Within the downregulated genes, it was found that multiple genes had a statistically significant downregulation when compared to the total PCB levels. When left unadjusted, nine genes were found to be significantly under-expressed at average fold change < −1 or > 1 and p-value 0.05 in relation to total PCB concentration (Table 3). After adjusting for maternal age and sex of the child, two genes (*RPS4Y1* (p= <0.001), and *EIF1AY* (p= <0.001)) were found to be significantly downregulated. Fig. 3 graphically represents the linear relationships between the significantly up- and down-related genes in relation to total PCB levels.

### 3.4. Ingenuity pathway analysis of differentially expressed genes

**Fig. 4.**
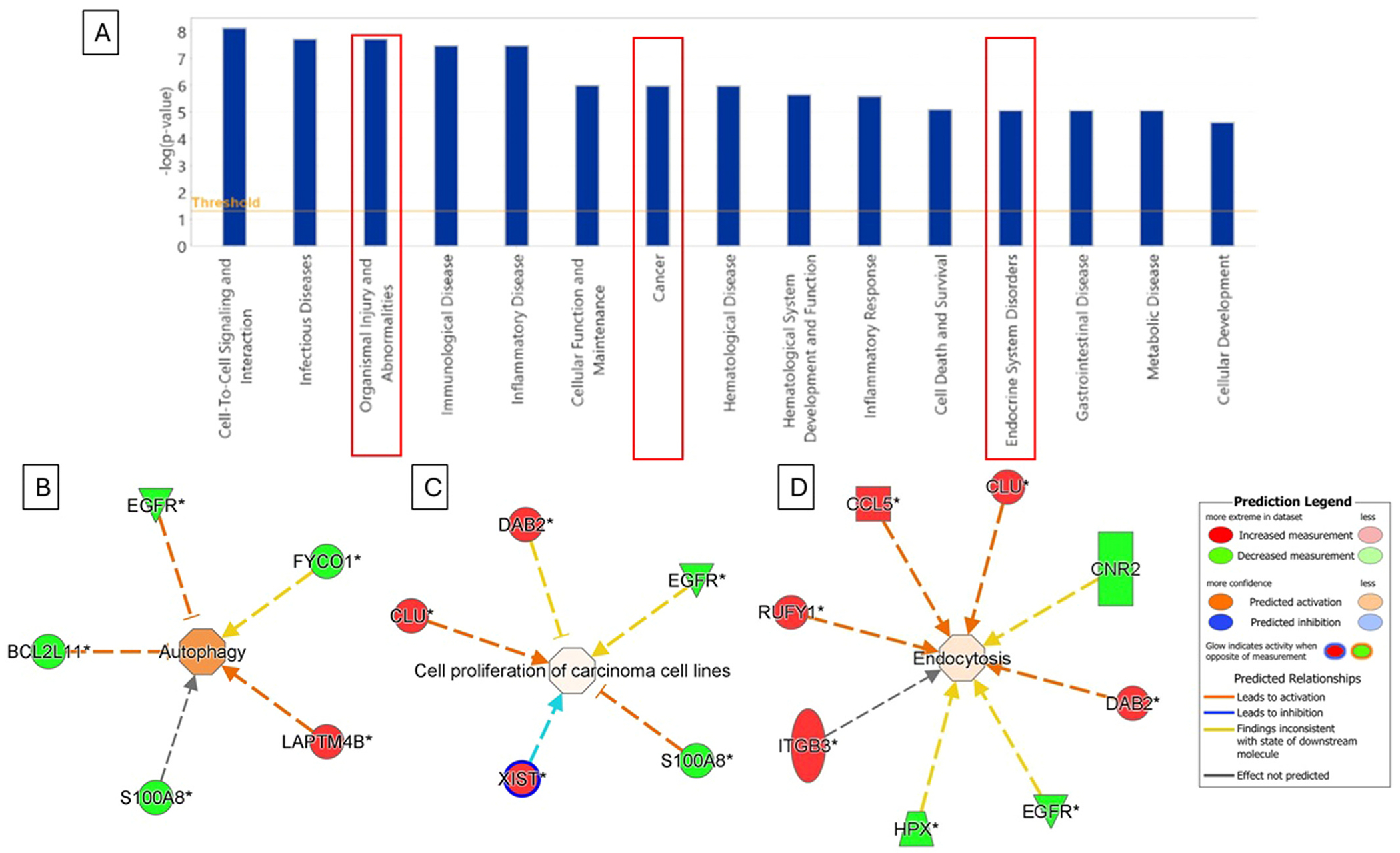
Ingenuity pathway analysis (IPA): A. **A.** Bar chart of significant diseases and disorders observed in cord blood (CB) samples. The Y-axis represents −log (P-value), with a threshold value of 1.5. Diseases and disorders marked within the red box indicate findings similar to those reported in our previous cohort from the 45-month study group (Mondal et al., 2022). **B.** Network of Autophagy (predicted activation). **C.** Network of cell proliferation in carcinoma cell lines (predicted activation). **D.** Network of Endocytosis (predicted activation). The construction of the network relied on information stored in the Ingenuity Pathways Knowledge Base (*IPKB*), along with actual expression data. Connections between differentially expressed genes were analyzed, focusing on those exhibiting a fold change of ≥ 1.5 and *p*-value < 0.05. Genes that are upregulated are represented by geometric figures in red, while downregulated genes are depicted in green. The intensity of the red and green colors reflects the degree of up- or downregulation, respectively, in the expression dataset.

IPA was performed to explore the potential biological pathways and disease processes associated with the differentially expressed genes identified in cord blood in relation to prenatal PCB exposure. The top enriched disease and functional categories (Fig. 4A) included Cell-to-Cell Signaling and Interaction, Infectious Diseases, Organismal Injury and Abnormalities, Cancer, Immunological Disease, Inflammatory Disease, Cellular Function and Maintenance, Hematological Disease, Cell Death and Survival, and Endocrine System Disorders. The highest significance was observed for Cell-to-Cell Signaling and Interaction, followed by Infectious Diseases and Organismal Injury and Abnormalities. Categories related to Cancer and Endocrine System Disorders were also strongly enriched, indicating potential relevance of PCB-associated transcriptional alterations to tumorigenic and hormonal regulatory processes. IPA functional network analysis highlighted several key molecular interactions (Fig. 4B–D). Autophagy network (Fig. 4B) included *EGFR, BCL2L11, FYCO1, LAPTM4B*, and *S100A8*, with predicted activation or inhibition relationships. Notably, *EGFR* and *BCL2L11* were downregulated, while *FYCO1* was upregulated, suggesting altered autophagy regulation in PCB-exposed newborns. Cell proliferation of carcinoma cell lines (Fig. 4C) involved *DAB2, CLU, EGFR, S100A8*, and *XIST*. IPA predicted complex modulation, with *DAB2* and *CLU* upregulated, while *XIST* was significantly downregulated, potentially influencing cell growth pathways relevant to carcinogenesis. Endocytosis network (Fig. 4D) encompassed *CCL5, CLU, CNR2, DAB2, EGFR, HPX, ITGB3*, and *RUFY1*. Multiple genes (*CCL5, CLU, DAB2, ITGB3, RUFY1*) were upregulated, indicating possible enhancement of endocytic processes and vesicular trafficking.

### 3.5. Effects of sex and maternal age at delivery on gene expression

The stratified results for the males and females are shown in Supplementary Table 3 and 4. Within males, multiple genes that were found to have a statistically significant fold change in relation to total PCB. When left unadjusted, two genes were found to have statistically significant at average fold change < −1 or > 1 and p-value < 0.05 within males (Table 4). For example, these genes included RUFY1 (p= 0.036, avg fold change= 1.486), and ELOVL7 (p= 0.036, avg fold change= 1.011). When adjusting for maternal age, it was found that no genes had a statistically significant fold change within males.

Within females, it was found that multiple genes had a statistically significant fold change in relation to total PCB. When left unadjusted, nine genes were found to have statistically significant at average fold change < −1 or > 1 and p-value 0.05: some examples include FXYD3 (p= 0.020, avg fold change=−1.721), DEFB124 (p= 0.030, avg fold change=−1.533), and *DAB2* (p= 0.048, avg fold change=1.426). When adjusting for maternal age, it was found that two genes had a statistically significant fold change within females: DAB2 (p= 0.009), and S100A8 (p= 0.016) (Table 4 and Supplementary Table 4).

An additional nonparametric analysis using the Wilcoxon rank-sum (Mann–Whitney U) test for male vs. female gene expression levels in cord blood identified six genes (RPS4Y1, EIF1AY, XIST, S100A8, EXOC6B, and PAM) that were significantly differentially expressed, both with and without the inclusion of outliers (Supplementary Table 15).

## 4. Discussion

### 4.1. Overview of the findings

In this study, we aimed to explore relationships between PCB levels and gene expression levels in maternal and cord blood, from a study of environmentally exposed mothers and infants in Slovakia. PCB levels in maternal and cord blood were highly correlated, supporting our decision to focus the molecular study on the cord blood specimens, and all of the specific PCB congeners we examined showed this same pattern of maternal-infant similarity. This study extends our previous work in the Slovak PCB-exposed cohort in several important ways (Ghosh et al., 2015; Mondal et al., 2022). We focus on newborns at delivery, rather than older children, providing a snapshot of transcriptional changes at the very onset of postnatal life. By analyzing paired maternal and cord blood samples, we link maternal exposures directly to newborn molecular signatures. Our microarray approach captures a broader transcriptional landscape than the targeted panels used in earlier studies.

Regarding gene expression results in cord blood, we observed that gene expression was associated in a linear fashion with increasing levels of total PCB. Specifically, gene over-expression (upregulation) trend was observed for 10 genes, four of which remained statistically significant after adjustment for maternal age and infant sex. XIST1 and EXOC6B exemplified this set of results. Conversely, under-expression (downregulation) in relation to total PCB concentration was observed for nine genes, among which two remained statistically significant after taking maternal age and infant sex into account. Exemplars of downregulated genes were EIF1AY and RPS4Y1.

Some differences in gene expression were observed in male infants compared to females. Male-associated expression was detected for RUFY1 (upregulated) and ELOVL7 (upregulated), whereas females showed significant downregulation in FXYD3, DEFB124, and upregulation in DAB2 in relation to total PCB concentration.

Ingenuity Pathway Analysis provided mechanistic insight into the potential biological effects of prenatal PCB exposure. The top enriched categories Cell-to-Cell Signaling and Interaction, Infectious Diseases, Organismal Injury and Abnormalities, Cancer, and Endocrine System Disorders, align with epidemiologic evidence linking PCBs to immune dysregulation, carcinogenesis, and endocrine disruption. Notably, the enrichment of Cancer and Endocrine System Disorders pathways is consistent with prior reports in PCB-exposed populations, where altered hormone signaling and tumor-promoting molecular changes have been observed (Ghosh et al., 2015; 2018).

### 4.2. Comparison of our results with prior research on PCBs

Previous studies on the Eastern Slovak population have investigated PCB levels in mother’s and cord blood. This population is known to have high PCB exposure due to environmental contamination, as documented in multiple other studies (Jusko et al., 2011; Park et al., 2010). Jusko et al. (2011) found that, for six PCB congeners, the median sum of cord blood PCBs was 343 ng/g lipid and in mother’s blood it was 455 ng/g lipid. Another study reported that maternal PCB concentrations were generally higher than cord levels (Park et al., 2010), which we observed as well in our sample.

Other studies in this region have investigated cord blood PCB exposure effects on child development. Park et al. (2010) found that higher concentrations of mono-ortho-substituted PCBs in the mother’s blood were associated with lower scores on childhood neurodevelopmental tests. Park et al. (2009) similarly found that higher concentrations of the metabolite 4-OH-CB-107 in cord blood were linked to reductions in psychomotor and mental development indices at 16 months. However, Simeone et al. (2022) did not find significant associations between PCB levels and neurodevelopment at 45 months. These findings, in general, emphasize the potential for prenatal PCB exposure to influence early childhood development.

Our prior research in this population has also focused on gene expression changes in older children. Dutta et al. (2010) found that higher PCB exposure among 45-month children was significantly linked to the differential expression of the genes BCL2, PON1, and ITGB1, which we did not observe in the present study of newborns. These genes are linked to biological pathways including cell-to-cell signaling and xenobiotic metabolism. Similarly, Ghosh et al. (2018) identified associations of PCB exposure in the 45-month-old children with potential cancer development pathway genes, while an earlier report (Ghosh et al., 2015) observed associations with metabolic, endocrine, and developmental pathways.

### 4.3. Functional importance of the significantly altered genes in the life course

Our global expression analysis of cord blood samples identified significant downregulation of XIST, and upregulation of EXOC6B, EIF1AY, and RPS4Y1. This our first study linking prenatal PCBs to XIST dysregulation in newborns, suggesting sex-chromosome impacts. XIST is a long non-coding RNA essential for X-chromosome inactivation (XCI), an epigenetic process ensuring dosage compensation of X-linked genes in females (Brown et al., 1992; Carrel and Willard, 2005). Downregulation XCI has been linked to developmental disorders, skewed XCI syndromes, X-linked diseases, and cancer (Plath et al., 2003; McHugh et al., 2015). In newborns, aberrant expression of XIST has been implicated in the development of bronchopulmonary dysplasia (BPD), a chronic lung disease primarily affecting premature infants. A study by Yuan et al. (2020) demonstrated that silencing XIST in newborn mice suppressed BPD development. The mechanism involves XIST binding to micro-RNA-101–3p, leading to the downregulation of high-mobility group protein B3 (HMGB3) and modulation of the transforming growth factor-beta 1 (TGF-β1)/Smad3 signaling pathway. This pathway is known to play a significant role in lung development and fibrosis (Yuan et al., 2020). Additionally, improper XIST expression can affect the regulation of X-linked genes. For instance, in the context of somatic cell nuclear transfer (SCNT) embryos, abnormal expression of XIST has been shown to impair development by inappropriately silencing X-linked genes. RNA interference-mediated knockdown of XIST in these embryos rescued the impaired development, highlighting the importance of precise XIST regulation for normal gene expression and development (Matoba et al., 2011).

EXOC6B, a critical exocyst complex component, facilitates synaptic vesicle transport and supports brain growth (Evers et al., 2014). Dysregulation has been associated with neurodevelopmental delays and synaptic dysfunction (He and Guo, 2009; Mei and Guo, 2018). Research indicates that EXOC6B plays a significant role in maintaining pancreatic β-cell function. A study demonstrated that silencing EXOC6B in rat pancreatic β-cells led to impaired insulin secretion, reduced insulin content, and disrupted exocytosis machinery. These findings suggest that EXOC6B is vital for proper insulin release and β-cell functionality (Sulaiman et al., 2022). Additionally, the disruption of EXOC6B has been associated with developmental delay and epilepsy. A case study identified a patient with developmental delay and epilepsy who had a disruption in the EXOC6B gene due to a de novo balanced translocation. This suggests that proper functioning of EXOC6B is important for normal neurological development (Frühmesser et al., 2013).

EIF1AY, a Y-linked gene, stabilizes the pre-initiation complex for protein synthesis. Its reduced expression disrupts protein production and cell proliferation, contributing to male-specific embryonic abnormalities (Pottmeier et al., 2024). RPS4Y1 encodes a ribosomal protein critical for mRNA translation and ribosome biogenesis. Dysregulation impairs cell growth and is linked to Turner syndrome (Chen et al., 2021).

The functional networks highlight plausible mechanistic routes by which PCB exposure may contribute to disease susceptibility. n the cancer-related network, upregulation of DAB2 and CLU alongside marked XIST downregulation (−80.58-fold) suggests disruption of genomic stability and tumor-suppressive pathways (Carrel and Willard, 2005; Yang et al., 2021). The endocrine-related network involved EGFR and ITGB3, which regulate growth factor signaling and tissue homeostasis (Normanno et al., 2006), processes known to be PCB-sensitive (Pocar et al., 2001). Within the organismal injury category, altered BCL2L11 and FYCO1 expression points to dysregulated apoptosis and autophagy key mechanisms for tissue repair and immune defense (O’Reilly et al., 2000; Pankiv et al., 2010). Changes in endocytosis-related genes (CCL5, RUFY1) may impact immune cell trafficking (Rot and von Andrian, 2004).

Our gender-based analysis revealed dysregulation of additional genes, including RUFY1, ELOVL7, S100A8, FXYD3, DEFB124, and DAB2. RUFY1 is key in antigen presentation and lysosome pathways, with dysregulation linked to immune dysfunction. ELOVL7, critical for very long-chain fatty acid (VLCFA) synthesis, impacts lipid metabolism, brain development, and myelination, with dysregulation contributing to neurological disorders. S100A8 is a protein, specifically a calcium-binding protein, that plays a role in inflammation and immune responses (Wang et al., 2018). FXYD3 affects ion transport and homeostasis, with implications in cancer progression (Li et al., 2023). *DEFB124* is essential for antimicrobial defense, and its dysregulation increases infection susceptibility (Kim et al., 2014). DAB2, crucial for endocytosis and signal transduction, has been linked to cancer progression and contains key functional domains which allow it to negatively regulate key signaling pathways including the mitogen activated protein kinase (MAPK), wingless/integrated (Wnt) and transforming growth factor beta (TGFβ) pathways (Price et al., 2022).

These findings provide mechanistic hypotheses on how prenatal PCB exposure may contribute to developmental and disease-related processes. However, we acknowledge that gene expression profiles in cord blood represent a single snapshot at birth and may not directly translate to long-term phenotypic outcomes without longitudinal follow-up. Therefore, our functional interpretations are preliminary and should be viewed as hypothesis-generating. Future prospective studies, following exposed children into later life, will be essential to determine whether these early transcriptional changes have sustained biological or clinical relevance.

### 4.4. Strengths and limitations of our study

Strengths of this study, which is among the first to report on maternal-infant pairs exposed to PCBs and the relationships of these exposures to gene expression, include the rigor of the experimental methods of nucleic acid extractions and measurements of gene expression levels. DNA and RNA extraction were standardized and quantified according to well-established protocols, and transcriptomic gene expression was quantified by the use of HG-U133 Plus 2.0 array. The GeneChip (HG-U133A 2.0) includes more than 54,000 probe sets used to analyze the expression levels of over 47,000 transcripts and variants, including approximately 38,500 well-characterized human genes. To ensure quality control in the global gene expression array, several measures were implemented. These included maintaining a false positive percentage of < 5 % and a significance level of 0.05, > 4-fold cRNA amplification from total RNA/cDNA, scaling factors kept below 2 to achieve whole-chip normalization of 800, and visual inspection of hybridization patterns to identify any chip defects. On the other hand, this was a pilot study with a small sample size, which may have generated results that cannot be generalized to the entire cohort of exposed mothers and infants in the study region. A limitation of our sex-stratified analyses is the small number of male and female participants, which reduces statistical power and may explain why no significant genes were detected in males after age adjustment. Accordingly, our sex-specific results should be considered exploratory in nature. These preliminary findings provide hypothesis-generating observations that warrant validation in larger, independent studies to more definitively establish sex-related transcriptional differences associated with prenatal PCB exposure.

We also acknowledge the limitations of this study which is the lack of independent validation of microarray findings using quantitative PCR (qPCR) or RNA-sequencing (RNA-seq). While the Affymetrix Human Genome U133 Plus 2.0 platform provides broad transcriptome coverage, it is known to have limited sensitivity for low-abundance transcripts and can be prone to technical variability, especially when detecting genes with large fold changes. Our observed expression levels of *XIST* and *EIF1AY*, although statistically significant, showed unusually high fold-changes that warrant cautious interpretation. Unfortunately, due to the restricted volume of available biospecimens and the absence of additional samples from this birth cohort, we were unable to perform experimental validation. We also acknowledge that while maternal age and newborn sex were adjusted for in our analyses, other potentially relevant maternal factors, including BMI, socioeconomic status, and smoking status, were not available in sufficient detail for the present cohort. The absence of these variables is a limitation, as they may influence gene expression patterns and newborn outcomes. Future studies with expanded clinical and demographic data collection will be essential to more comprehensively evaluate the impact of these potential confounders.

## 5. Conclusions

The effects of environmental PCB exposures on human health have been investigated for decades, yet there continue to be gaps in knowledge on their effects on child health and development, especially for the newborn infant. Our results in this pilot study suggest that alterations in gene expression are already observable in cord blood, and it is interesting to speculate on their possible consequences as the child develops and matures. Alterations in key pathways such as neurodevelopment and tumorigenesis should be further investigated in prospective studies in this and other PCB-exposed cohorts to more fully define the legacy of environmental PCB contamination.

## Supplementary Material

1

## Figures and Tables

**Table 1:** Demographic characteristics of the gene-expression cohort and molecular study subgroup.

		Gene Expression Cohort	Molecular Study Subgroup	P-Value
**Age of mother at delivery**				
	*N*	*76*	*24*	
	Mean (± SD)	25.9 (± 4.1)	25.7 (± 4.2)	
	Median	26	25.5	
	Min-Max	18–35	18–33	
**Has the mother lived her whole life in the region? n (%)**				0.907
	*N*	*76*	*24*	
	Yes	61 (80.3 %)	19 (79.2 %)	
	No	15 (19.7 %)	5 (20.8 %)	
	If not, mean years lived in the region.	2.3	2.6	
**Type of Delivery, n (%)**				0.616
	*N*	*76*	*24*	
	Spontaneous, Vaginal	72 (94.7 %)	23(95.8 %)	
	Instrumental	0 (0 %)	0 (0 %)	
	Cesarean Section	4(5.3 %)	1(4.2 %)	
**Newborn Birth Weight, (g)**				0.423
	*N*	*91*	*29*	
	Mean (± SD)	3412.1 (± 451.30)	3486.6 (± 375.75)	
	Median	3400	3500	
	Min-Max	2340–4550	2850–4550	
**Sex of Newborn, n (%)**				0.741
	*N*	*91*	*29*	
	Female	44 (48.4 %)	13 (44.8 %)	
	Male	47 (51.6 %)	16 (55.2 %)	

**Table 2:** PCB concentration in mother’s blood (MB) and cord blood (CB) for the gene-expression cohort and molecular study subgroup.

	Gene expression cohort	Molecular study subgroup	P-Value
	*(N = 91)*	*(N = 29)*	
**Total MB PCB Concentration**			0.188
Mean (± SD)	685.96 (± 839.18)	947.92 (± 1168.60)	
Median	421.68	394.37	
Min-Max	86.61–5921.70	123.87–5921.70	
**Total CB PCB Concentration**			0.034
Mean (± SD)	519.13 (± 617.66)	841.39 (± 923.43)	
Median	287.8	368.57	
Min-Max	58.72–4113.51	75.48–4113.51	

**Table 3:** Genes expression in fold change in relation to total PCB concentration, organized by highest to lowest fold change.

	Change in Gene Expression per Increase in Total PCB per nanogram per gram lipid	Unadjusted P-Value	Average Fold Change	Adjusted P-Value
**Genes With Upregulation Trend**				
DAB2	0.00051	0.0321	1.809	0.189
ITGB3	0.00096	0.0199	1.623	0.126
TPGS2	0.00053	0.0186	1.595	0.273
EXOC6B	0.00091	0.0263	1.419	0.034
RUFY1	0.00062	0.007	1.412	0.107
DNM3	0.00063	0.043	1.408	0.077
C2orf88	0.00073	0.0333	1.381	0.172
PAM	0.0008	0.0256	1.339	0.039
CLU	0.00076	0.0135	1.081	0.037
XIST	0.01327	0.0207	−13.912	< 0.001
**Genes With Downregulation Trend**				
FXYD3	−0.00038	0.0317	−1.888	0.318
HCG22	−0.00052	0.0196	−1.744	0.265
EGFR	−0.00059	0.0337	−1.679	0.148
RNF19B	−0.00068	0.0277	−1.455	0.186
LINC00520	−0.0004	0.047	−1.25	0.264
HPX	−0.0004	0.0474	−1.246	0.24
CNR2	−0.00066	0.0372	−1.066	0.412
RPS4Y1	−0.01406	0.0092	15.726	< 0.001
EIF1AY	−0.02581	0.0076	78.861	< 0.001

Note: EXOC6B, CLU, XIST, RPS4Y1, and EIF1AY remained significant after adjustment.

**Table 4:** Average fold changes of males versus females.

	Change in Gene Expression per Increase in Total PCB per nanogram per gram lipid	Unadjusted P-Value	Average Fold Change	Adjusted P-Value*
***Genes in Males***				
RUFY1	0.0007	0.036	1.486	0.134
ELOVL7	0.0009	0.036	1.011	0.152
***Genes in Females***				
DAB2	0.0033	0.048	1.426	0.009
TPGS2	0.0014	0.006	1.384	0.137
RUFY1	0.0009	0.015	1.296	0.269
S100A8	−0.0036	0.003	1.161	0.016
IGHD	−0.0017	0.019	−1.412	0.113
C19orf84	−0.003	0.032	−1.205	0.116
RNF19B	−0.0036	0.081	−0.735	0.593
FXYD3	−0.0023	0.02	−1.721	0.387
DEFB124	−0.0038	0.03	−1.533	0.272

* Adjustment to P-Value was based on the maternal age and sex of the baby

## Data Availability

Data will be made available on request.

## Data sharing statement

Data are stored and may be available upon reasonable request complying with the current data sharing policy of NIH, available at https://grants.nih. The data sets used in this study include personal information. Thus, datasets (Coded and with no personal information) are available from the corresponding author, Dr. Somiranjan Ghosh, on reasonable requests. All raw and processed transcriptomic data generated in this study is available in the NCBI Gene Expression Omnibus (GEO) database (GSE32420) upon acceptance of the manuscript, to ensure public accessibility, transparency, and reproducibility of the findings.

## Appendix A. Supporting information

Supplementary data associated with this article can be found in the online version at doi:10.1016/j.ecoenv.2025.119059.

## Abbreviations:

PCBs: Polychlorinated Biphenyls
MB: Mother’s Blood
CB: Cord Blood
HRGC/HRMS: High Resolution Gas Chromatography/High Resolution Mass Spectrometry
PAXgene: PreAnalytiX Gene Tubes
TAC: Transcriptome Analysis Console
GC-RMA: GC Robust Multi-array Average
SAS: Statistical Analysis Software
SD: Standard Deviation
ANOVA: Analysis of Variance
HCH: Hexachlorocyclohexane
HCB: Hexachlorobenzene
BMI: Body Mass Index
DDT: Dichlorodiphenyltrichloroethane
DDE: Dichlorodiphenyldichloroethylene
SCNT: Somatic Cell Nuclear Transfer
VLCFA: Very Long-Chain Fatty Acid
IPA: Ingenuity Pathway Analysis
CRNA: Complementary RNA
RNAi: RNA interference
LncRNA: Long Non-Coding RNA
