# Supplementary material for: Exploring the associations between prenatal PCB exposures and gene expression: Observations from a study of newborn Slovak infants
Source: Ecotoxicol Environ Saf. Author manuscript; Available in PMC 2025 Oct 27. (PMC12557832; doi:10.1016/j.ecoenv.2025.119059)

**Appendix A. Supplementary data**

Supplementary Figure 1: Individual Participant CB Concentration for Molecular Study Subgroup, arranged from lowest to highest exposure


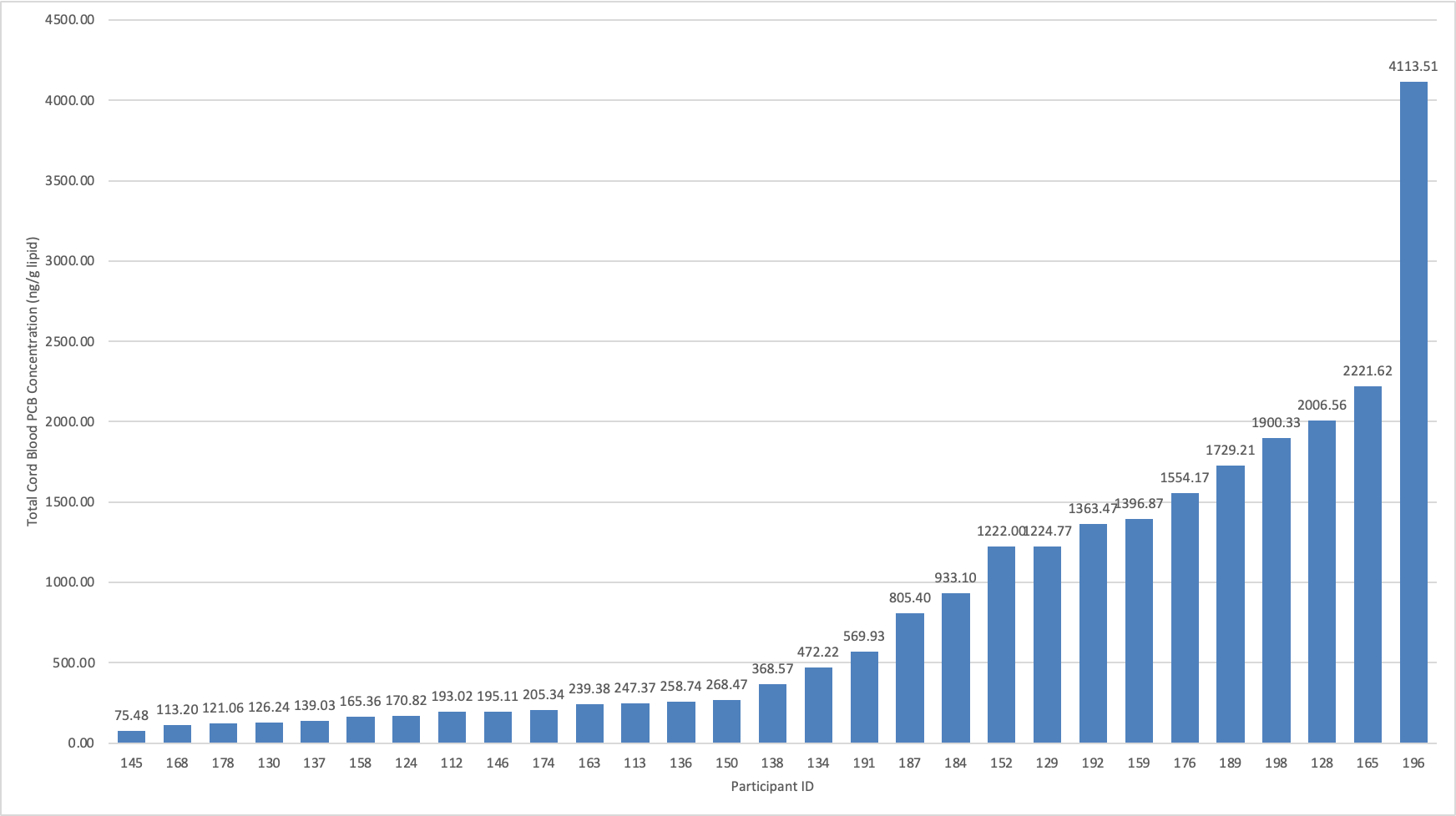


Supplementary Figure 2. Individual Participant MB Concentration for Molecular Study Subgroup, arranged from lowest to highest exposure


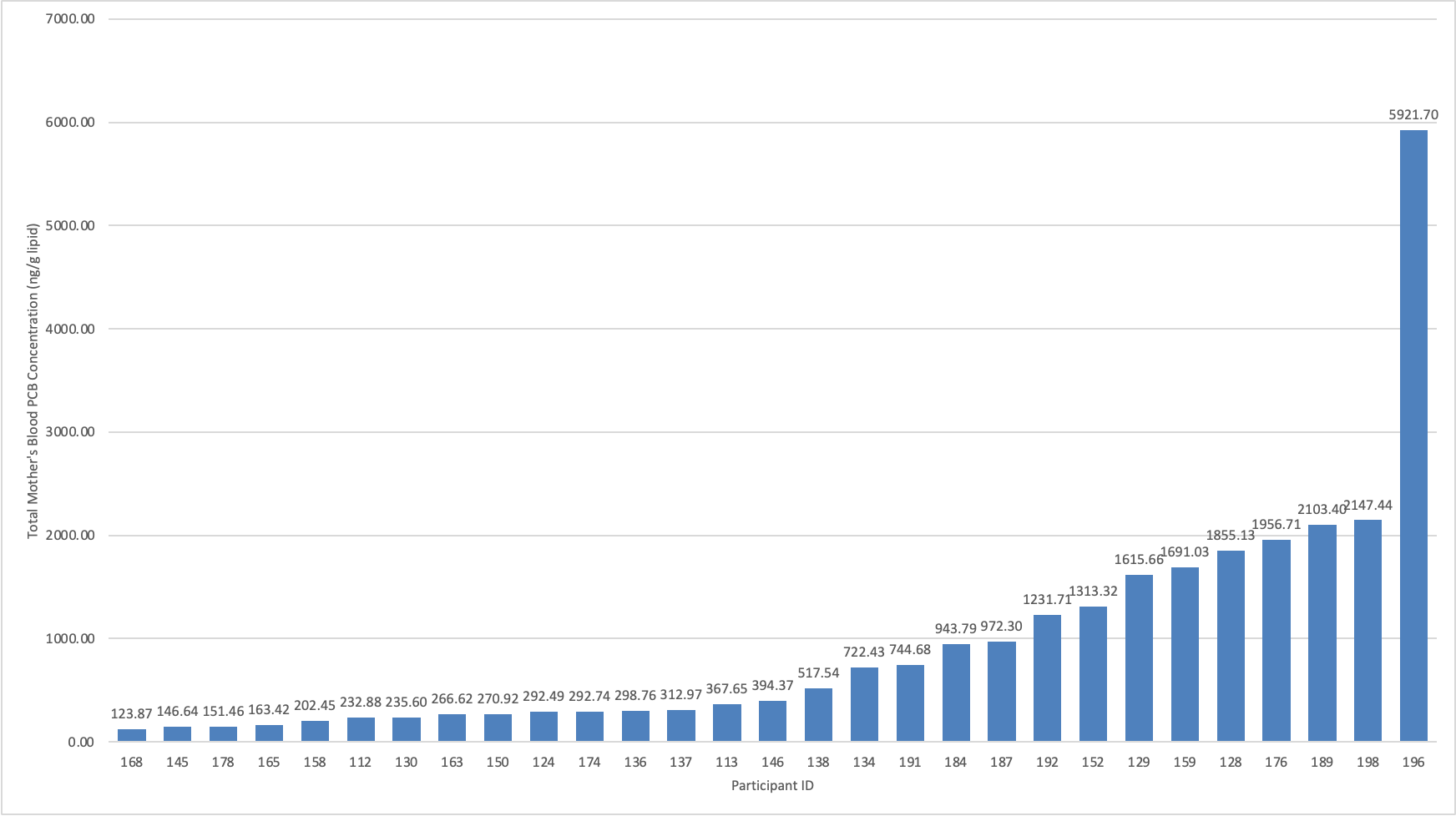


Supplementary Figure 3. Fold Anomaly XIST - 224588_at Graphical Representation

*
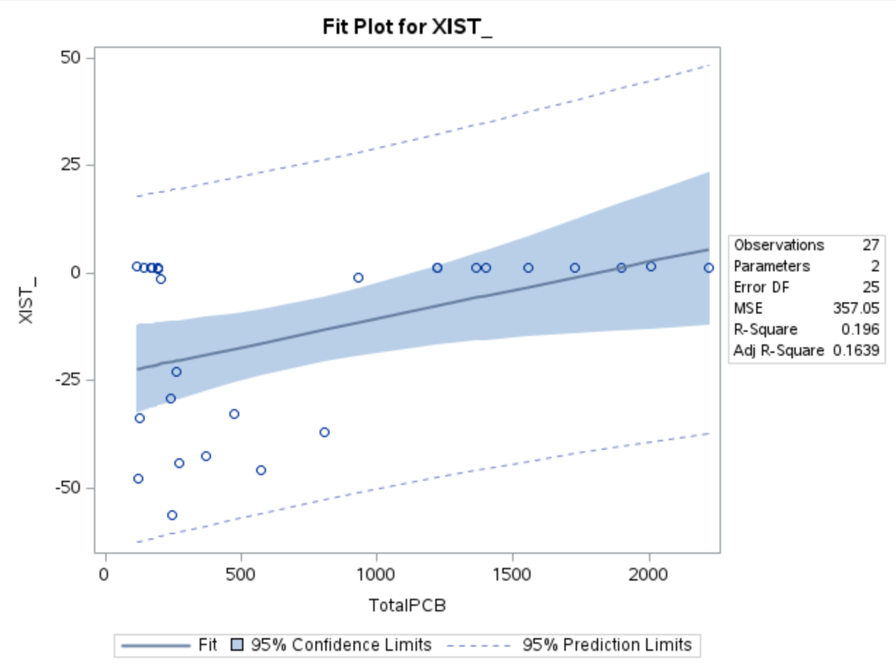
*

| Supplementary Table 1: Genes With Upregulation in Fold Change Based on Total PCB Concentration, Organized by Highest to Lowest Fold Change | | | | | | | | |  |  |
| --- | --- | --- | --- | --- | --- | --- | --- | --- | --- | --- |
|  |  |  |  |  |  |  |  |  |  |  |
| Genes | | Change in Gene Expression per Increase in Total PCB per nanogram per gram lipid | | Unadjusted P-Value | Average Fold Change | | Adjusted P-Value | |  |  |
|  | |  | |  |  | |  | |  |  |
| 242990_AT | | 0.00048 | | 0.2509 | 2.178 | | 0.446 | |  |  |
| 227501_AT | | 0.00078 | | 0.0288 | 1.836 | | 0.16 | |  |  |
| DAB2: 201280_s_at | | 0.00051 | | 0.0321 | 1.809 | | 0.189 | |  |  |
| ITGB3: 204627_s_at | | 0.00096 | | 0.0199 | 1.623 | | 0.126 | |  |  |
| CCL5: 1555759_a_at | | 0.00066 | | 0.0745 | 1.614 | | 0.61 | |  |  |
| TPGS2: 213616_at | | 0.00053 | | 0.0186 | 1.595 | | 0.273 | |  |  |
| 242403_AT | | 0.00043 | | 0.2469 | 1.577 | | 0.228 | |  |  |
| EXOC6B: 225900_at | | 0.00091 | | 0.0263 | 1.419 | | 0.034 | |  |  |
| RUFY1: 218243_at | | 0.00062 | | 0.007 | 1.412 | | 0.107 | |  |  |
| DNM3: 209839_at | | 0.00063 | | 0.043 | 1.408 | | 0.077 | |  |  |
| C2orf88: 228195_at | | 0.00073 | | 0.0333 | 1.381 | | 0.172 | |  |  |
| PAM: 202336_s_at | | 0.0008 | | 0.0256 | 1.339 | | 0.039 | |  |  |
| PAM: 212958_x_at | | 0.00064 | | 0.1328 | 1.338 | | 0.639 | |  |  |
| TAOK3: 220761_s_at | | 0.00028 | | 0.3076 | 1.283 | | 0.846 | |  |  |
| CLU: 208791_at | | 0.00076 | | 0.0135 | 1.081 | | 0.037 | |  |  |
| ZKSCAN1: 1557953_at | | 0.00053 | | 0.1238 | 0.974 | | 0.442 | |  |  |
| CLEC1B: 220496_at | | 0.00062 | | 0.0984 | 0.893 | | 0.654 | |  |  |
| CD9: 201005_at | | 0.00084 | | 0.0388 | 0.75 | | 0.229 | |  |  |
| LAPTM4B: 214039_s_at | | 0.00077 | | 0.0445 | 0.742 | | 0.232 | |  |  |
| TUBB1: 230690_at | | 0.00087 | | 0.0259 | 0.679 | | 0.311 | |  |  |
| ELOVL7: 227180_at | | 0.00103 | | 0.0172 | 0.66 | | 0.205 | |  |  |
| GNG11: 204115_at | | 0.00106 | | 0.0141 | 0.539 | | 0.218 | |  |  |
| HIST1H2BG: 210387_at | | 0.00084 | | 0.0882 | 0.505 | | 0.421 | |  |  |
| ZNF281: 222619_at | | 0.00094 | | 0.0232 | 0.376 | | 0.221 | |  |  |
| BAIAP2 AS1: 1566557_at | | 0.00118 | | 0.0042 | 0.191 | | 0.061 | |  |  |
| SKAP2: 216899_s_at | | 0.00099 | | 0.0316 | 0.112 | | 0.309 | |  |  |
| DLK1: 209560_s_at | | 0.00142 | | 0.0227 | -0.226 | | 0.267 | |  |  |
| XIST:  227671_at | | 0.01327 | | 0.0207 | -13.912 | | <0.001 | |  |  |
| XIST: 224588_at | | 0.07354 | | 0.0319 | -80.58 | | <0.001 | |  |  |
|  | |  | |  |  | |  | |  |  |
| Supplementary Table 2: Gene Down-Regulation Fold Change Based on Total PCB Concentration, Organized by Lowest to Highest Fold Change | | | | | | | | | | |
| *Genes* | | Change in Gene Expression per Increase in Total PCB per nanogram per gram lipid | | Unadjusted P-Value | | | Average Fold Change | | Adjusted P-Value | |
|  | |  | |  | | |  | |  | |
| DEFB124: 1568377_x_at | | -0.00046 | | 0.0691 | | | -1.937 | | 0.485 | |
| FXYD3: 202488_s_at | | -0.00038 | | 0.0317 | | | -1.888 | | 0.318 | |
| HCG22: 1560767_at | | -0.00052 | | 0.0196 | | | -1.744 | | 0.265 | |
| 1568513 X AT | | -0.00065 | | 0.0301 | | | -1.691 | | 0.253 | |
| EGFR: 1565483_at | | -0.00059 | | 0.0337 | | | -1.679 | | 0.148 | |
| IGHD: 215621_s_at | | -0.00031 | | 0.155 | | | -1.551 | | 0.435 | |
| RNF19B: 36564_at | | -0.00068 | | 0.0277 | | | -1.455 | | 0.186 | |
| C19orf84: 1560830_a_at | | -0.00046 | | 0.052 | | | -1.439 | | 0.236 | |
| 1568649_AT | | -0.00048 | | 0.0753 | | | -1.36 | | 0.304 | |
| ZNF548: 1553718_at | | -0.00048 | | 0.0786 | | | -1.341 | | 0.484 | |
| LINC00520: 1555786_s_at | | -0.0004 | | 0.047 | | | -1.25 | | 0.264 | |
| HPX: 39763_at | | -0.0004 | | 0.0474 | | | -1.246 | | 0.24 | |
| BCL2L11: 1553096_s_at | | -0.00052 | | 0.059 | | | -1.116 | | 0.318 | |
| LINC01210: 1556507_at | | -0.00064 | | 0.0794 | | | -1.102 | | 0.18 | |
| CNR2: 206586_at | | -0.00066 | | 0.0372 | | | -1.066 | | 0.412 | |
| LINC00491: 1564281_at | | -0.00075 | | 0.0517 | | | -1.037 | | 0.388 | |
| LMF1: 1569871_at | | -0.00092 | | 0.0169 | | | -0.705 | | 0.337 | |
| PVRL2: 232078_at | | -0.00078 | | 0.0363 | | | -0.38 | | 0.474 | |
| FYCO1: 1555523_a_at | | -0.00088 | | 0.0165 | | | -0.165 | | 0.33 | |
| S100A8: 214370_at | | -0.0008 | | 0.0604 | | | 0.049 | | 0.241 | |
| RPS4Y1: 201909_at | | -0.01406 | | 0.0092 | | | 15.726 | | <0.001 | |
| EIF1AY: 204410_at | | -0.07052 | | 0.0104 | | | 27.848 | | <0.001 | |
| EIF1AY: 204409_s_at | | -0.02581 | | 0.0076 | | | 78.861 | | <0.001 | |

| Supplementary Table 3: Average Fold Change of Males | | | | |
| --- | --- | --- | --- | --- |
| *Genes* | Change in Gene Expression per Increase in Total PCB per nanogram per gram lipid | Unadjusted P-Value | Average Fold Change | Adjusted P-Value |
|  |  |  |  |  |
| 242990_AT | 0.0001 | 0.813 | 2.591 | 0.959 |
| 227501_AT | 0.0003 | 0.133 | 2.385 | 0.385 |
| EXOC6B: 225900_at | 0.0003 | 0.235 | 2.166 | 0.475 |
| ITGB3: 204627_s_at | 0.0006 | 0.12 | 2.118 | 0.137 |
| 242403_AT | -0.00003 | 0.912 | 2.087 | 0.929 |
| DAB2: 201280_s_at | 0.0003 | 0.164 | 2.036 | 0.419 |
| CCL5: 1555759_a_at | 0.0004 | 0.282 | 1.877 | 0.544 |
| C2orf88: 228195_at | 0.0003 | 0.207 | 1.836 | 0.544 |
| PAM: 212958_x_at | 0.0004 | 0.21 | 1.779 | 0.187 |
| DNM3: 209839_at | 0.0003 | 0.135 | 1.751 | 0.182 |
| TPGS2: 213616_at | 0.0005 | 0.108 | 1.711 | 0.306 |
| PAM: 202336_s_at | 0.0002 | 0.558 | 1.615 | 0.586 |
| RUFY1: 218243_at | 0.0007 | 0.036 | 1.486 | 0.134 |
| TAOK3: 220761_s_at | 0.0002 | 0.601 | 1.408 | 0.83 |
| CLU: 208791_at | 0.0006 | 0.07 | 1.335 | 0.111 |
| ZKSCAN1: 1557953_at | 0.0002 | 0.405 | 1.284 | 0.729 |
| CD9: 201005_at | 0.0006 | 0.181 | 1.1 | 0.493 |
| CLEC1B: 220496_at | 0.0006 | 0.172 | 1.078 | 0.439 |
| ELOVL7: 227180_at | 0.0009 | 0.036 | 1.011 | 0.152 |
| XIST: 224588_at | 0.0002 | 0.418 | 0.988 | 0.641 |
| LAPTM4B: 214039_s_at | 0.0007 | 0.101 | 0.935 | 0.326 |
| HIST1H2BG: 210387_at | 0.0004 | 0.388 | 0.916 | 0.507 |
| TUBB1: 230690_at | 0.0009 | 0.057 | 0.876 | 0.196 |
| GNG11: 204115_at | 0.001 | 0.049 | 0.85 | 0.169 |
| BAIAP2 AS1: 1566557_at | 0.001 | 0.038 | 0.641 | 0.151 |
| SKAP2: 216899_s_at | 0.0006 | 0.215 | 0.639 | 0.387 |
| ZNF281: 222619_at | 0.0009 | 0.062 | 0.557 | 0.212 |
| RPS4Y1: 201909_at | -0.0928 | 0.275 | 0.428 | 0.234 |
| DLK1: 209560_s_at | 0.0012 | 0.083 | 0.269 | 0.273 |
| EIF1AY: 204410_at | 0.0006 | 0.22 | -0.405 | 0.453 |
| XIST:  227671_at | -0.0001 | 0.754 | -0.469 | 0.786 |
| FYCO1: 1555523_a_at | -0.0007 | 0.087 | -0.47 | 0.298 |
| S100A8: 214370_at | -0.0002 | 0.575 | -0.541 | 0.768 |
| PVRL2: 232078_at | -0.0006 | 0.117 | -0.654 | 0.363 |
| EIF1AY: 204409_s_at | 0.0006 | 0.204 | -0.749 | 0.434 |
| LMF1: 1569871_at | -0.0006 | 0.134 | -1.093 | 0.169 |
| LINC00491: 1564281_at | -0.0006 | 0.154 | -1.26 | 0.19 |
| HPX: 39763_at | -0.0003 | 0.111 | -1.341 | 0.213 |
| LINC01210: 1556507_at | -0.0004 | 0.295 | -1.4 | 0.153 |
| CNR2: 206586_at | -0.0004 | 0.051 | -1.414 | 0.179 |
| BCL2L11: 1553096_s_at | -0.0001 | 0.177 | -1.478 | 0.316 |
| LINC00520: 1555786_s_at | -0.0002 | 0.09 | -1.494 | 0.299 |
| ZNF548: 1553718_at | -0.0003 | 0.173 | -1.501 | 0.441 |
| IGHD: 215621_s_at | -0.0004 | 0.169 | -1.551 | 0.369 |
| C19orf84: 1560830_a_at | 0.0004 | 0.153 | -1.553 | 0.232 |
| 1568649_AT | -0.0002 | 0.19 | -1.7 | 0.466 |
| EGFR: 1565483_at | -0.0005 | 0.114 | -1.841 | 0.136 |
| RNF19B: 36564_at | -0.0003 | 0.239 | -1.869 | 0.346 |
| 1568513 X AT | -0.0005 | 0.148 | -1.9 | 0.255 |
| FXYD3: 202488_s_at | -0.0003 | 0.059 | -1.948 | 0.207 |
| HCG22: 1560767_at | -0.0003 | 0.083 | -1.97 | 0.276 |
| DEFB124: 1568377_x_at | -0.0002 | 0.258 | -2.145 | 0.384 |
| *** Adjustment to P-Value was based on the maternal age and sex of the baby* | | | | |

| Supplementary Table 4: Average Fold Change of Females | | | | | | |  |
| --- | --- | --- | --- | --- | --- | --- | --- |
| *Genes* | Change in Gene Expression per Increase in Total PCB per nanogram per gram lipid | | Unadjusted P-Value | | Average Fold Change | Adjusted P-Value |  |
|  |  | |  | |  |  |  |
| 242990_AT | 0.0019 | | 0.479 | | 1.433 | 0.957 |  |
| 227501_AT | 0.0019 | | 0.483 | | 0.917 | 0.962 |  |
| EXOC6B: 225900_at | 0.0024 | | 0.403 | | 0.196 | 0.768 |  |
| ITGB3: 204627_s_at | 0.0032 | | 0.255 | | 0.763 | 0.615 |  |
| 242403_AT | 0.0005 | | 0.833 | | 0.728 | 0.999 |  |
| DAB2: 201280_s_at | 0.0033 | | 0.048 | | 1.426 | 0.009 |  |
| CCL5: 1555759_a_at | 0.003 | | 0.174 | | 1.161 | 0.719 |  |
| C2orf88: 228195_at | 0.004 | | 0.113 | | 0.595 | 0.322 |  |
| PAM: 212958_x_at | 0.0036 | | 0.131 | | 0.517 | 0.389 |  |
| DNM3: 209839_at | 0.0039 | | 0.101 | | 0.78 | 0.229 |  |
| TPGS2: 213616_at | 0.0014 | | 0.006 | | 1.384 | 0.137 |  |
| PAM: 202336_s_at | 0.0072 | | 0.004 | | 0.85 | 0.078 |  |
| RUFY1: 218243_at | 0.0009 | | 0.015 | | 1.296 | 0.269 |  |
| TAOK3: 220761_s_at | 0.0015 | | 0.257 | | 1.013 | 0.667 |  |
| CLU: 208791_at | 0.0032 | | 0.112 | | 0.658 | 0.039 |  |
| ZKSCAN1: 1557953_at | 0.002 | | 0.404 | | 0.419 | 0.74 |  |
| CD9: 201005_at | 0.0036 | | 0.149 | | 0.114 | 0.196 |  |
| CLEC1B: 220496_at | 0.0023 | | 0.371 | | 0.537 | 0.805 |  |
| ELOVL7: 227180_at | 0.003 | | 0.328 | | -0.01 | 0.314 |  |
| XIST: 224588_at | -0.0206 | | 0.448 | | -34.352 | 0.974 |  |
| LAPTM4B: 214039_s_at | 0.0033 | | 0.186 | | 0.359 | 0.546 |  |
| HIST1H2BG: 210387_at | 0.0054 | | 0.071 | | -0.26 | 0.647 |  |
| TUBB1: 230690_at | 0.0035 | | 0.148 | | 0.255 | 0.405 |  |
| GNG11: 204115_at | 0.0031 | | 0.252 | | -0.036 | 0.288 |  |
| BAIAP2 AS1: 1566557_at | 0.0035 | | 0.091 | | -0.692 | 0.177 |  |
| SKAP2: 216899_s_at | 0.0032 | | 0.234 | | -0.833 | 0.688 |  |
| ZNF281: 222619_at | 0.0036 | | 0.13 | | 0.007 | 0.534 |  |
| RPS4Y1: 201909_at | -0.0128 | | 0.433 | | 37.198 | 0.846 |  |
| DLK1: 209560_s_at | 0.0043 | | 0.255 | | -1.193 | 0.435 |  |
| EIF1AY: 204410_at | -0.0256 | | 0.182 | | 69.795 | 0.868 |  |
| XIST:  227671_at | 0.085 | | 0.687 | | -205.759 | 0.643 |  |
| FYCO1: 1555523_a_at | -0.0038 | | 0.069 | | 0.434 | 0.65 |  |
| S100A8: 214370_at | -0.0036 | | 0.003 | | 1.161 | 0.016 |  |
| PVRL2: 232078_at | -0.003 | | 0.186 | | 0.179 | 0.737 |  |
| EIF1AY: 204409_s_at | 0.0334 | | 0.611 | | 195.66 | 0.133 |  |
| LMF1: 1569871_at | -0.0052 | | 0.018 | | 0.017 | 0.228 |  |
| LINC00491: 1564281_at | -0.0048 | | 0.049 | | -0.581 | 0.581 |  |
| HPX: 39763_at | -0.0019 | | 0.144 | | -1.065 | 0.45 |  |
| LINC01210: 1556507_at | -0.0033 | | 0.178 | | -0.563 | 0.707 |  |
| CNR2: 206586_at | -0.001 | | 0.661 | | -0.381 | 0.977 |  |
| BCL2L11: 1553096_s_at | -0.0031 | | 0.133 | | -0.438 | 0.092 |  |
| LINC00520: 1555786_s_at | -0.0023 | | 0.164 | | -0.834 | 0.621 |  |
| ZNF548: 1553718_at | -0.0029 | | 0.122 | | -0.997 | 0.491 |  |
| IGHD: 215621_s_at | -0.0017 | | 0.019 | | -1.412 | 0.113 |  |
| C19orf84: 1560830_a_at | -0.003 | | 0.032 | | -1.205 | 0.116 |  |
| 1568649_AT | -0.002 | | 0.338 | | -0.732 | 0.944 |  |
| EGFR: 1565483_at | -0.0026 | | 0.114 | | -1.365 | 0.535 |  |
| RNF19B: 36564_at | -0.0036 | | 0.081 | | -0.735 | 0.593 |  |
| 1568513 X AT | -0.0035 | | 0.029 | | -1.284 | 0.213 |  |
| FXYD3: 202488_s_at | -0.0023 | | 0.02 | | -1.721 | 0.387 |  |
| HCG22: 1560767_at | -0.0028 | | 0.073 | | -1.338 | 0.425 |  |
| DEFB124: 1568377_x_at | -0.0038 | | 0.03 | | -1.533 | 0.272 |  |
| *** Adjustment to P-Value was based on the maternal age and sex of the baby* | | | | | | |  |
| *Supplementary Table 5: Genes Average Fold Change Based on Total PCB Concentration and Mothers Age, Sorted by P-Value Highest to Lowest* | | | | | | |  |
| Genes | | Unadjusted P-Value | | Change in Gene Expression per Increase in Total PCB per nanogram per gram lipid | | | |
|  | |  | |  | | | |
| CLU: 208791_at | | 0.013 | | 0.0005 | | | |
| BAIAP2 AS1: 1566557_at | | 0.032 | | 0.0012 | | | |
| PAM: 212958_x_at | | 0.05 | | 0.0008 | | | |
| RUFY1: 218243_at | | 0.05 | | 0.0005 | | | |
| EGFR: 1565483_at | | 0.079 | | -0.0005 | | | |
| ITGB3: 204627_s_at | | 0.081 | | 0.0006 | | | |
| ELOVL7: 227180_at | | 0.094 | | 0.0008 | | | |
| LINC01210: 1556507_at | | 0.099 | | -0.0006 | | | |
| DAB2: 201280_s_at | | 0.101 | | 0.0003 | | | |
| DNM3: 209839_at | | 0.103 | | 0.0005 | | | |
| ZNF281: 222619_at | | 0.105 | | 0.0009 | | | |
| GNG11: 204115_at | | 0.106 | | 0.0009 | | | |
| XIST: 224588_at | | 0.117 | | 0.0126 | | | |
| EIF1AY: 204409_s_at | | 0.126 | | -0.0609 | | | |
| LAPTM4B: 214039_s_at | | 0.129 | | 0.0008 | | | |
| HCG22: 1560767_at | | 0.13 | | -0.0003 | | | |
| DLK1: 209560_s_at | | 0.131 | | 0.0012 | | | |
| EIF1AY: 204410_at | | 0.135 | | -0.0195 | | | |
| 1568513 X AT | | 0.138 | | -0.0005 | | | |
| RPS4Y1: 201909_at | | 0.148 | | -0.0109 | | | |
| C19orf84: 1560830_a_at | | 0.15 | | -0.0004 | | | |
| TPGS2: 213616_at | | 0.15 | | 0.0005 | | | |
| CD9: 201005_at | | 0.163 | | 0.0008 | | | |
| TUBB1: 230690_at | | 0.165 | | 0.0008 | | | |
| HPX: 39763_at | | 0.17 | | -0.0002 | | | |
| FYCO1: 1555523_a_at | | 0.172 | | -0.0007 | | | |
| LMF1: 1569871_at | | 0.176 | | -0.0007 | | | |
| RNF19B: 36564_at | | 0.179 | | -0.0006 | | | |
| XIST:  227671_at | | 0.184 | | 0.0652 | | | |
| SKAP2: 216899_s_at | | 0.201 | | 0.0008 | | | |
| C2orf88: 228195_at | | 0.208 | | 0.0005 | | | |
| FXYD3: 202488_s_at | | 0.219 | | -0.0003 | | | |
| LINC00491: 1564281_at | | 0.233 | | -0.0006 | | | |
| EXOC6B: 225900_at | | 0.241 | | 0.0007 | | | |
| 227501_AT | | 0.248 | | 0.0006 | | | |
| CNR2: 206586_at | | 0.259 | | -0.0005 | | | |
| LINC00520: 1555786_s_at | | 0.261 | | -0.0002 | | | |
| PVRL2: 232078_at | | 0.277 | | -0.0006 | | | |
| S100A8: 214370_at | | 0.277 | | -0.0007 | | | |
| DEFB124: 1568377_x_at | | 0.285 | | -0.0003 | | | |
| HIST1H2BG: 210387_at | | 0.306 | | 0.0005 | | | |
| BCL2L11: 1553096_s_at | | 0.35 | | -0.0003 | | | |
| 1568649_AT | | 0.384 | | -0.0003 | | | |
| CCL5: 1555759_a_at | | 0.418 | | 0.0004 | | | |
| IGHD: 215621_s_at | | 0.423 | | -0.0002 | | | |
| ZKSCAN1: 1557953_at | | 0.434 | | 0.0004 | | | |
| PAM: 202336_s_at | | 0.44 | | 0.0005 | | | |
| CLEC1B: 220496_at | | 0.444 | | 0.0004 | | | |
| ZNF548: 1553718_at | | 0.471 | | -0.0002 | | | |
| 242990_AT | | 0.701 | | 0.0004 | | | |
| TAOK3: 220761_s_at | | 0.71 | | 0.0002 | | | |
| 242403_AT | | 0.807 | | 0.0002 | | | |

| *Supplementary Table 6. MB PCB Concentrations for All Congeners in Molecular Study Subgroup* | | | | | | |
| --- | --- | --- | --- | --- | --- | --- |
|  | *N* | Mean | SD | Median | Max | Min |
| PCB74 | *29* | 14.18 | 15.56 | 9.57 | 66.01 | 1.68 |
| PCB66 | *27* | 2.55 | 2.54 | 1.85 | 11.09 | 0.17 |
| PCB99 | *29* | 5.4 | 4.7 | 3.75 | 20.09 | 0.82 |
| PCB118 | *29* | 15.96 | 25.71 | 9.59 | 142.06 | 2.5 |
| PCB105 | *29* | 3.26 | 5.43 | 1.72 | 27.89 | 0.34 |
| PCB146 | *29* | 17.72 | 18.27 | 9.84 | 71.5 | 1.74 |
| PCB153 | *29* | 219.15 | 220.36 | 120.52 | 960.45 | 27.75 |
| PCB138 | *29* | 120.04 | 162.16 | 66.61 | 839.91 | 14.59 |
| PCB167 | *29* | 6.41 | 12.7 | 3 | 69.92 | 0.9 |
| PCB156 | *29* | 18.61 | 29.61 | 7.96 | 160.61 | 0.31 |
| PCB157 | *29* | 1.84 | 3.74 | 0.87 | 20.66 | 0.27 |
| PCB178 | *29* | 12.15 | 12.18 | 4.93 | 37.76 | 1.13 |
| PCB187 | *29* | 26.81 | 29.79 | 10.43 | 115.88 | 2.04 |
| PCB183 | *29* | 10.59 | 12.73 | 4.41 | 57.94 | 1 |
| PCB177 | *29* | 9.22 | 12.26 | 4.04 | 57.2 | 0.07 |
| PCB171 | *29* | 5.7 | 6.64 | 3.4 | 25.42 | 0.51 |
| PCB172 | *29* | 5.25 | 6.66 | 2.69 | 24.48 | 0.24 |
| PCB180 | *29* | 225.43 | 294.58 | 94.96 | 1505.05 | 12.67 |
| PCB170 | *29* | 104.24 | 150.56 | 45.12 | 795.39 | 7.14 |
| PCB189 | *29* | 3.05 | 5.88 | 1.01 | 31.97 | 0.18 |
| PCB202 | *29* | 3.83 | 3.92 | 1.83 | 11.73 | 0.29 |
| PCB199 | *29* | 46.77 | 64.77 | 17.78 | 316.96 | 1.94 |
| PCB196 | *29* | 28.3 | 40.27 | 11.71 | 208.82 | 1.41 |
| PCB195 | *29* | 10.07 | 16.64 | 3.67 | 88.42 | 0.42 |
| PCB194 | *29* | 28.34 | 51.38 | 10.85 | 276.72 | 1.01 |
| PCB206 | *29* | 2.97 | 7.24 | 1.15 | 39.75 | 0.19 |
| PCB209 | *28* | 0.28 | 0.52 | 0.15 | 2.81 | 0.03 |
| Total PCB | *29* | 947.92 | 1168.6 | 394.37 | 5921.7 | 123.87 |
| *All highlighted rows indicate selected PCB Congeners for analysis | | | | | | |

| *Supplementary Table 7. MB PCB Concentrations for Selected Congeners in Molecular Study Subgroup* | | | | | | |
| --- | --- | --- | --- | --- | --- | --- |
|  | *N* | Mean | SD | Median | Max | Min |
| PCB118 | *29* | 15.96 | 25.71 | 9.59 | 142.06 | 2.5 |
| PCB105 | *29* | 3.26 | 5.43 | 1.72 | 27.89 | 0.34 |
| PCB138 | *29* | 120.04 | 162.16 | 66.61 | 839.91 | 14.59 |
| PCB153 | *29* | 219.15 | 220.36 | 120.52 | 960.45 | 27.75 |
| PCB156 | *29* | 18.61 | 29.61 | 7.96 | 160.61 | 0.31 |
| PCB157 | *29* | 1.84 | 3.74 | 0.87 | 20.66 | 0.27 |
| PCB180 | *29* | 225.43 | 294.58 | 94.96 | 1505.05 | 12.67 |
| PCB170 | *29* | 104.24 | 150.56 | 45.12 | 795.39 | 7.14 |
| PCB189 | *29* | 3.05 | 5.88 | 1.01 | 31.97 | 0.18 |
| Total PCB | *29* | 947.92 | 1168.6 | 394.37 | 5921.7 | 123.87 |

| *Supplementary Table 8. CB PCB Concentrations for Selected Congeners in Molecular Study Subgroup* | | | | | | |
| --- | --- | --- | --- | --- | --- | --- |
|  | *N* | Mean | SD | Median | Max | Min |
| PCB118 | *29* | 22.7 | 48.28 | 8.75 | 235.89 | 0.41 |
| PCB105 | *22* | 7.22 | 21.74 | 1.32 | 101.24 | 0.28 |
| PCB138 | *29* | 115.87 | 145.86 | 62.72 | 681.44 | 14.68 |
| PCB153 | *29* | 194.93 | 187.36 | 116.57 | 687.95 | 20.6 |
| PCB156 | *29* | 15.23 | 22.86 | 5.83 | 121.85 | 0.13 |
| PCB157 | *28* | 1.76 | 3.28 | 0.8 | 17.56 | 0.03 |
| PCB180 | *29* | 186.07 | 199.48 | 97.76 | 909.12 | 6.72 |
| PCB170 | *29* | 86.64 | 113.26 | 42.79 | 577.83 | 3.07 |
| PCB189 | *29* | 2.29 | 3.84 | 0.84 | 20.57 | 0.03 |
| TotalPCB | *29* | 841.39 | 923.43 | 368.57 | 4113.51 | 75.48 |

| *Supplementary Table 9. MB PCB High vs. Low Concentration for Molecular Study Subgroup* | | | | | |
| --- | --- | --- | --- | --- | --- |
|  |  |  | PCB Level < 394.37 | PCB Level > 394.37 |  |
|  |  |  | *(N = 14)* | *(N = 15)* | *P-Value* |
| Age of mother at delivery |  |  |  |  | 0.0124 |
|  | *N* |  | *13* | *11* |  |
|  | Mean (± SD) |  | 23.8 (± 3.6) | 27.9 (± 3.8) |  |
|  | Median |  | 22 | 29 |  |
|  | Min-Max |  | 18-32 | 21-23 |  |
| Has the mother lived her whole life in the region? n (%) |  |  |  |  | 0.6987 |
|  | *N* |  | *13* | *11* |  |
|  | Yes |  | 10 (76.9%) | 9 (81.8%) |  |
|  | No |  | 3 (23.1%) | 2 (18.2%) |  |
|  | If No, mean years lived in the region. |  | 2.7 | 2.5 |  |
| Type of Delivery, n (%) |  |  |  |  | 0.254 |
|  | *N* |  | *13* | *11* |  |
|  | Spontaneous, Vaginal |  | 13 (100%) | 10 (90.9%) |  |
|  | Instrumental |  | 0 (0%) | 0 (0%) |  |
|  | Cesarean Section |  | 0 (0%) | 1 (9.1%) |  |
| Newborn Birth Weight (g) |  |  |  |  | 0.727 |
|  | *N* |  | *14* | *15* |  |
|  | Mean (± SD) |  | 3460.7 (± 422.9) | 3510.7 (339.1) |  |
|  | Median |  | 3390 | 3620 |  |
|  | Max-Min |  | 2850-4550 | 2880-4050 |  |
| Sex of Newborn, n (%) |  |  |  |  | 0.7682 |
|  | *N* |  | *14* | *15* |  |
|  | Female |  | 6 (42.9%) | 7 (46.7%) |  |
|  | Male |  | 8 (57.1%) | 8 (53.3%) |  |

| *Supplementary Table 10. CB PCB High vs. Low Concentration for Molecular Study Subgroup* | | | | | | | | | | |
| --- | --- | --- | --- | --- | --- | --- | --- | --- | --- | --- |
|  | |  | |  | | PCB Level < 368.57 | | PCB Level > 368.57 | |  |
|  | |  | |  | | *(N = 14)* | | *(N = 15)* | | *P-Value* |
| Age of mother at delivery | |  | |  | |  | |  | | 0.022 |
|  | | *N* | |  | | *12* | | *12* | |  |
|  | | Mean (± SD) | |  | | 23.8 (± 3.8) | | 27.6 (± 3.8) | |  |
|  | | Median | |  | | 22 | | 28.5 | |  |
|  | | Min-Max | |  | | 18-32 | | 21-33 | |  |
| Has the mother lived her whole life in the region?, n (%) | |  | |  | |  | |  | | 0.505 |
|  | | *N* | |  | | *12* | | *12* | |  |
|  | | Yes | |  | | 9 (75%) | | 10 (83.3%) | |  |
|  | | No | |  | | 3 (25%) | | 2 (16.7%) | |  |
|  | | If No, mean years lived in the region. | |  | | 2.7 | | 2.5 | |  |
| Type of Delivery, n (%) | |  | |  | |  | |  | | 0.2963 |
|  | | *N* | |  | | *12* | | *12* | |  |
|  | | Spontaneous, Vaginal | |  | | 12 (100%) | | 11 (91.7%) | |  |
|  | | Instrumental | |  | | 0 (0%) | | 0 (0%) | |  |
|  | | Cesarean Section | |  | | 0 (0%) | | 1 (8.3%) | |  |
| Newborn Birth Weight (g) | |  | |  | |  | |  | | 0.535 |
|  | | *N* | |  | | *14* | | *15* | |  |
|  | | Mean (± SD) | |  | | 3440.7 (± 425.0) | | 3529.3 (± 332.5) | |  |
|  | | Median | |  | | 3365 | | 3620 | |  |
|  | | Max-Min | |  | | 2850-4550 | | 2880-4050 | |  |
| Sex of Newborn, n (%) | |  | |  | |  | |  | | 0.4386 |
|  | | *N* | |  | | *14* | | *15* | |  |
|  | | Female | |  | | 7 (50.0%) | | 6 (40.0%) | |  |
|  | | Male | |  | | 7 (50.0%) | | 9 (60.0%) | |  |
| *Supplementary Table 11. Genes Average Fold Change of Individuals Over Median Total PCB Concentration, Sorted by Average Fold Change Highest to Lowest* | | | | | | | | | |  |
| Gene | | Change in Gene Expression per Increase in Total PCB per nanogram per gram lipid | | Unadjusted P-Value | | Average Fold Change | | Adjusted P-Value | |  |
|  | |  | |  | |  | |  | |  |
| XIST: 224588_at | | 0.0921 | | 0.012 | | 126.863 | | 0.299 | |  |
| XIST:  227671_at | | 0.0061 | | 0.004 | | 6.574 | | 0.207 | |  |
| 242403_AT | | 0.0007 | | 0.288 | | 2.37 | | 0.2 | |  |
| PAM: 202336_s_at | | -0.0004 | | 0.576 | | 1.97 | | 0.798 | |  |
| CCL5: 1555759_a_at | | 0.0001 | | 0.833 | | 1.852 | | 0.575 | |  |
| EXOC6B: 225900_at | | 0.0011 | | 0.09 | | 1.817 | | 0.43 | |  |
| 242990_AT | | 0.0004 | | 0.619 | | 1.763 | | 0.377 | |  |
| 227501_AT | | 0.001 | | 0.074 | | 1.393 | | 0.153 | |  |
| ELOVL7: 227180_at | | 0.0004 | | 0.409 | | 1.379 | | 0.235 | |  |
| TAOK3: 220761_s_at | | 0.0003 | | 0.547 | | 1.259 | | 0.587 | |  |
| PAM: 212958_x_at | | 0.0003 | | 0.547 | | 1.173 | | 0.544 | |  |
| FYCO1: 1555523_a_at | | 0.00008 | | 0.816 | | 1.09 | | 0.244 | |  |
| CLEC1B: 220496_at | | 0.0002 | | 0.759 | | 0.843 | | 0.787 | |  |
| TPGS2: 213616_at | | 0.00009 | | 0.847 | | 0.822 | | 0.527 | |  |
| DEFB124: 1568377_x_at | | -0.0001 | | 0.662 | | 0.779 | | 0.907 | |  |
| C2orf88: 228195_at | | 0.001 | | 0.193 | | 0.776 | | 0.165 | |  |
| HIST1H2BG: 210387_at | | -0.0001 | | 0.862 | | 0.626 | | 0.418 | |  |
| ZNF281: 222619_at | | 0.001 | | 0.168 | | 0.61 | | 0.46 | |  |
| CLU: 208791_at | | 0.00005 | | 0.93 | | 0.273 | | 0.165 | |  |
| LINC01210: 1556507_at | | -0.0004 | | 0.518 | | 0.099 | | 0.867 | |  |
| RUFY1: 218243_at | | -0.00008 | | 0.891 | | 0.073 | | 0.079 | |  |
| TUBB1: 230690_at | | -0.0001 | | 0.779 | | 0.05 | | 0.998 | |  |
| LAPTM4B: 214039_s_at | | 0.0001 | | 0.829 | | -0.072 | | 0.829 | |  |
| DLK1: 209560_s_at | | 0.00008 | | 0.896 | | -0.32 | | 0.154 | |  |
| RNF19B: 36564_at | | -0.0014 | | 0.072 | | -0.426 | | 0.372 | |  |
| IGHD: 215621_s_at | | 0.0003 | | 0.646 | | -0.566 | | 0.712 | |  |
| ITGB3: 204627_s_at | | -0.0003 | | 0.463 | | -0.599 | | 0.572 | |  |
| S100A8: 214370_at | | 0.0009 | | 0.281 | | -0.606 | | 0.539 | |  |
| ZKSCAN1: 1557953_at | | -0.0006 | | 0.243 | | -0.613 | | 0.674 | |  |
| BCL2L11: 1553096_s_at | | 0.00008 | | 0.887 | | -0.661 | | 0.729 | |  |
| LMF1: 1569871_at | | -0.0002 | | 0.701 | | -0.668 | | 0.513 | |  |
| HPX: 39763_at | | -0.0001 | | 0.77 | | -0.737 | | 0.502 | |  |
| DNM3: 209839_at | | 0.00006 | | 0.886 | | -0.767 | | 0.207 | |  |
| SKAP2: 216899_s_at | | -0.0008 | | 0.068 | | -0.844 | | 0.319 | |  |
| CD9: 201005_at | | -0.001 | | 0.015 | | -0.91 | | 0.162 | |  |
| GNG11: 204115_at | | 0.0004 | | 0.187 | | -1.012 | | 0.698 | |  |
| BAIAP2 AS1: 1566557_at | | -0.0005 | | 0.106 | | -1.055 | | 0.146 | |  |
| C19orf84: 1560830_a_at | | -0.0005 | | 0.309 | | -1.164 | | 0.293 | |  |
| 1568649_AT | | -0.0007 | | 0.189 | | -1.23 | | 0.22 | |  |
| DAB2: 201280_s_at | | -0.0001 | | 0.4 | | -1.273 | | 0.513 | |  |
| LINC00520: 1555786_s_at | | -0.0006 | | 0.138 | | -1.311 | | 0.241 | |  |
| 1568513 X AT | | -0.0007 | | 0.251 | | -1.42 | | 0.335 | |  |
| LINC00491: 1564281_at | | -0.0001 | | 0.782 | | -1.6 | | 0.583 | |  |
| ZNF548: 1553718_at | | -0.00002 | | 0.921 | | -1.676 | | 0.934 | |  |
| EGFR: 1565483_at | | -0.0008 | | 0.102 | | -1.733 | | 0.331 | |  |
| CNR2: 206586_at | | -0.0003 | | 0.165 | | -1.772 | | 0.279 | |  |
| PVRL2: 232078_at | | -0.00025 | | 0.346 | | -1.792 | | 0.419 | |  |
| HCG22: 1560767_at | | -0.0002 | | 0.392 | | -1.89 | | 0.393 | |  |
| FXYD3: 202488_s_at | | -0.00006 | | 0.814 | | -1.916 | | 0.813 | |  |
| RPS4Y1: 201909_at | | -0.0162 | | 0.084 | | -26.13 | | 0.63 | |  |
| EIF1AY: 204410_at | | -0.0328 | | 0.115 | | -53.377 | | 0.647 | |  |
| EIF1AY: 204409_s_at | | -0.1242 | | 0.101 | | -192.033 | | 0.719 | |  |
| *** Adjustment to P-Value was based on the maternal age and sex of the baby* | | | | | | | | | |  |

| *Supplementary Table 12: Genes Average Fold Change of Individuals Under Median Total PCB Concentration* | | | | |
| --- | --- | --- | --- | --- |
| Gene | Change in Gene Expression per Increase in Total PCB per nanogram per gram lipid | Unadjusted P-Value | Average Fold Change | Adjusted P-Value |
|  |  |  |  |  |
| *XIST: 224588_at | -0.075 | 0.022 | 5.91 | <0.001 |
| XIST:  227671_at | -0.0498 | 0.061 | 4.101 | <0.001 |
| 242403_AT | -0.0042 | 0.541 | 1.999 | 0.79 |
| PAM: 202336_s_at | -0.0024 | 0.738 | 0.581 | 0.11 |
| CCL5: 1555759_a_at | -0.0027 | 0.651 | 0.64 | 0.892 |
| EXOC6B: 225900_at | 0.0013 | 0.802 | 0.0085 | 0.954 |
| 242990_AT | 0.0004 | 0.945 | 1.438 | 0.979 |
| 227501_AT | -0.0015 | 0.841 | -0.077 | 0.948 |
| ELOVL7: 227180_at | -0.0097 | 0.232 | -0.452 | 0.832 |
| TAOK3: 220761_s_at | -0.0046 | 0.417 | 0.627 | 0.333 |
| PAM: 212958_x_at | 0.0038 | 0.422 | 0.478 | 0.261 |
| FYCO1: 1555523_a_at | -0.008 | 0.055 | 0.699 | 0.516 |
| CLEC1B: 220496_at | -0.0085 | 0.233 | -0.464 | 0.908 |
| TPGS2: 213616_at | -0.0009 | 0.843 | 0.483 | 0.68 |
| DEFB124: 1568377_x_at | 0.0063 | 0.237 | -0.155 | 0.7 |
| C2orf88: 228195_at | 0.00005 | 0.994 | 0.228 | 0.67 |
| HIST1H2BG: 210387_at | -0.0057 | 0.487 | -1.007 | 0.947 |
| ZNF281: 222619_at | -0.0065 | 0.325 | -0.508 | 0.688 |
| CLU: 208791_at | -0.0022 | 0.672 | -0.556 | 0.967 |
| LINC01210: 1556507_at | 0.0022 | 0.618 | 0.659 | 0.965 |
| RUFY1: 218243_at | -0.0089 | 0.078 | 0.315 | 0.103 |
| TUBB1: 230690_at | 0.0014 | 0.672 | -0.99 | 0.785 |
| LAPTM4B: 214039_s_at | -0.0048 | 0.247 | -0.932 | 0.706 |
| DLK1: 209560_s_at | 0.0038 | 0.491 | 0.22 | 0.647 |
| RNF19B: 36564_at | 0.006 | 0.36 | -0.142 | 0.72 |
| IGHD: 215621_s_at | -0.0004 | 0.937 | -0.215 | 0.929 |
| ITGB3: 204627_s_at | 0.0078 | 0.179 | 0.0478 | 0.368 |
| S100A8: 214370_at | -0.0006 | 0.912 | -0.981 | 0.998 |
| ZKSCAN1: 1557953_at | -0.0036 | 0.393 | 1.333 | 0.641 |
| BCL2L11: 1553096_s_at | 0.0037 | 0.342 | -0.872 | 0.668 |
| LMF1: 1569871_at | 0.0012 | 0.824 | -0.321 | 0.853 |
| HPX: 39763_at | 0.005 | 0.383 | 0.348 | 0.662 |
| DNM3: 209839_at | 0.0006 | 0.892 | -0.548 | 0.976 |
| SKAP2: 216899_s_at | 0.0037 | 0.49 | 0.0164 | 0.767 |
| CD9: 201005_at | -0.0025 | 0.388 | -1.047 | 0.798 |
| GNG11: 204115_at | -0.0067 | 0.12 | -0.662 | 0.139 |
| BAIAP2 AS1: 1566557_at | -0.0042 | 0.151 | -0.992 | 0.339 |
| C19orf84: 1560830_a_at | 0.0052 | 0.402 | 0.0492 | 0.913 |
| 1568649_AT | 0.005 | 0.385 | -0.657 | 0.994 |
| DAB2: 201280_s_at | -0.0002 | 0.84 | -1.281 | 0.931 |
| LINC00520: 1555786_s_at | 0.00001 | 0.996 | -0.87 | 0.992 |
| 1568513 X AT | 0.0071 | 0.313 | -0.186 | 0.6 |
| LINC00491: 1564281_at | 0.0036 | 0.625 | -0.284 | 0.853 |
| ZNF548: 1553718_at | -0.001 | 0.851 | -0.92 | 0.674 |
| EGFR: 1565483_at | -0.0005 | 0.938 | -0.87 | 0.813 |
| CNR2: 206586_at | 0.0101 | 0.028 | -1.093 | 0.401 |
| PVRL2: 232078_at | 0.0093 | 0.023 | -1.029 | 0.144 |
| HCG22: 1560767_at | -0.0035 | 0.348 | -1.25 | 0.636 |
| FXYD3: 202488_s_at | -0.0051 | 0.373 | -0.948 | 0.24 |
| RPS4Y1: 201909_at | 0.1554 | 0.109 | -19.837 | <0.001 |
| *EIF1AY: 204410_at | 0.2876 | 0.163 | -38.371 | 0.001 |
| EIF1AY: 204409_s_at | 1.0966 | 0.116 | -133.272 | <0.001 |
| *** Adjustment to P-Value was based on the maternal age and sex of the baby* | | | | |

*Figures 9a and 9b: DISTRIBUTION CURVES FOR TOP TWO UP REGULATED GENES*

*
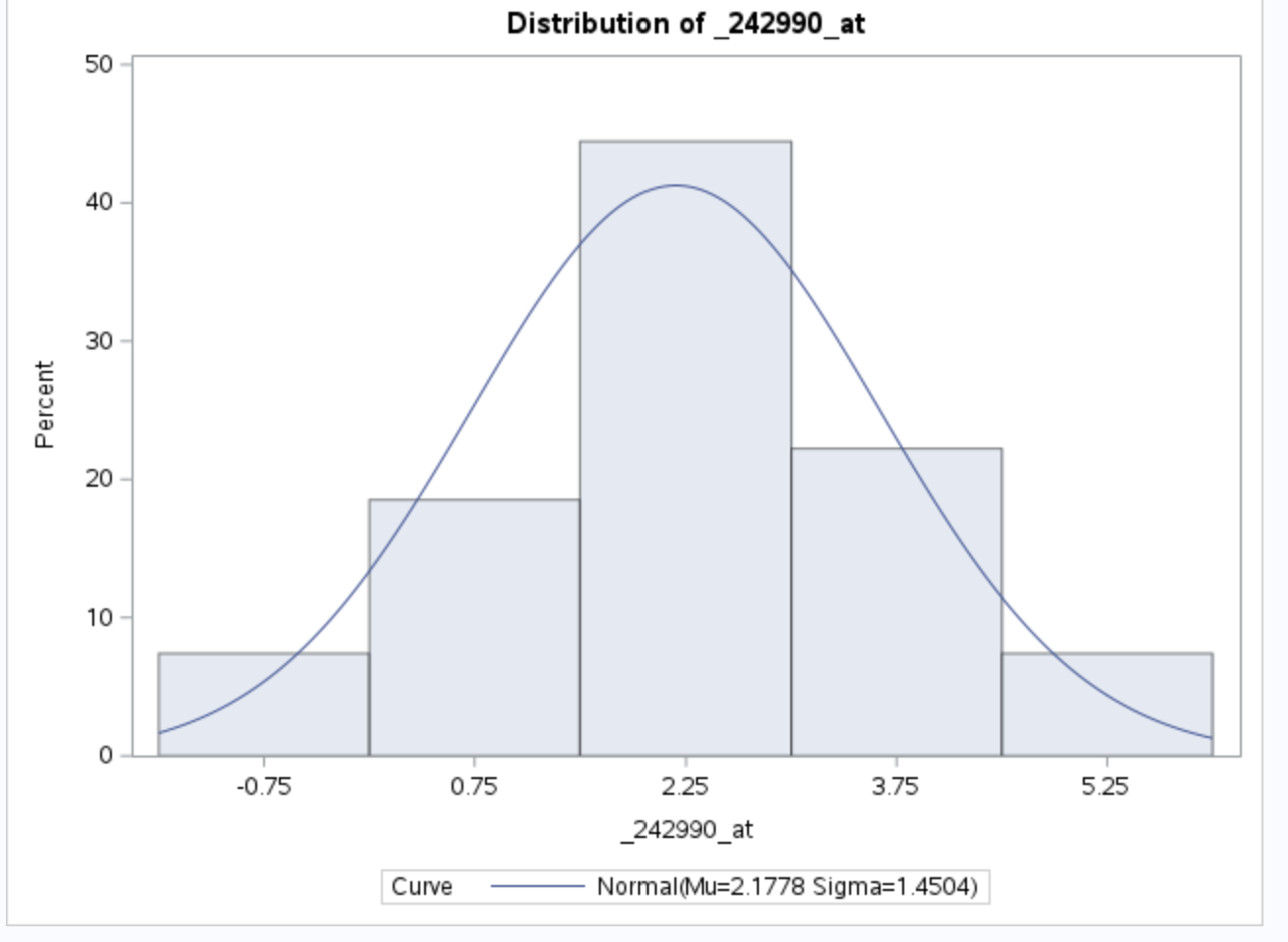
*

*
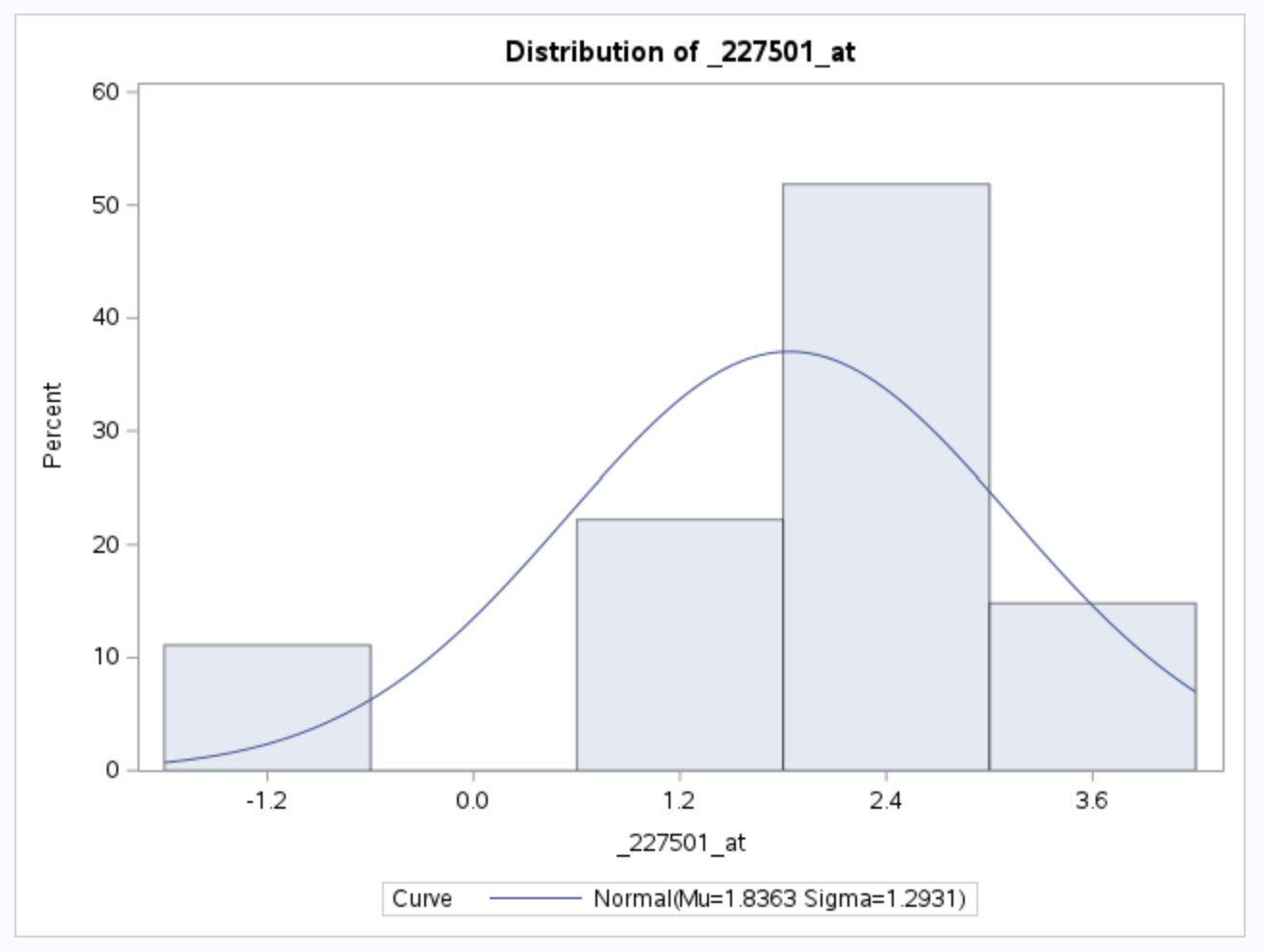
*

*Figures 10a and 10b: DISTRIBUTION CURVES FOR TOP TWO DOWN REGULATED GENES*

*
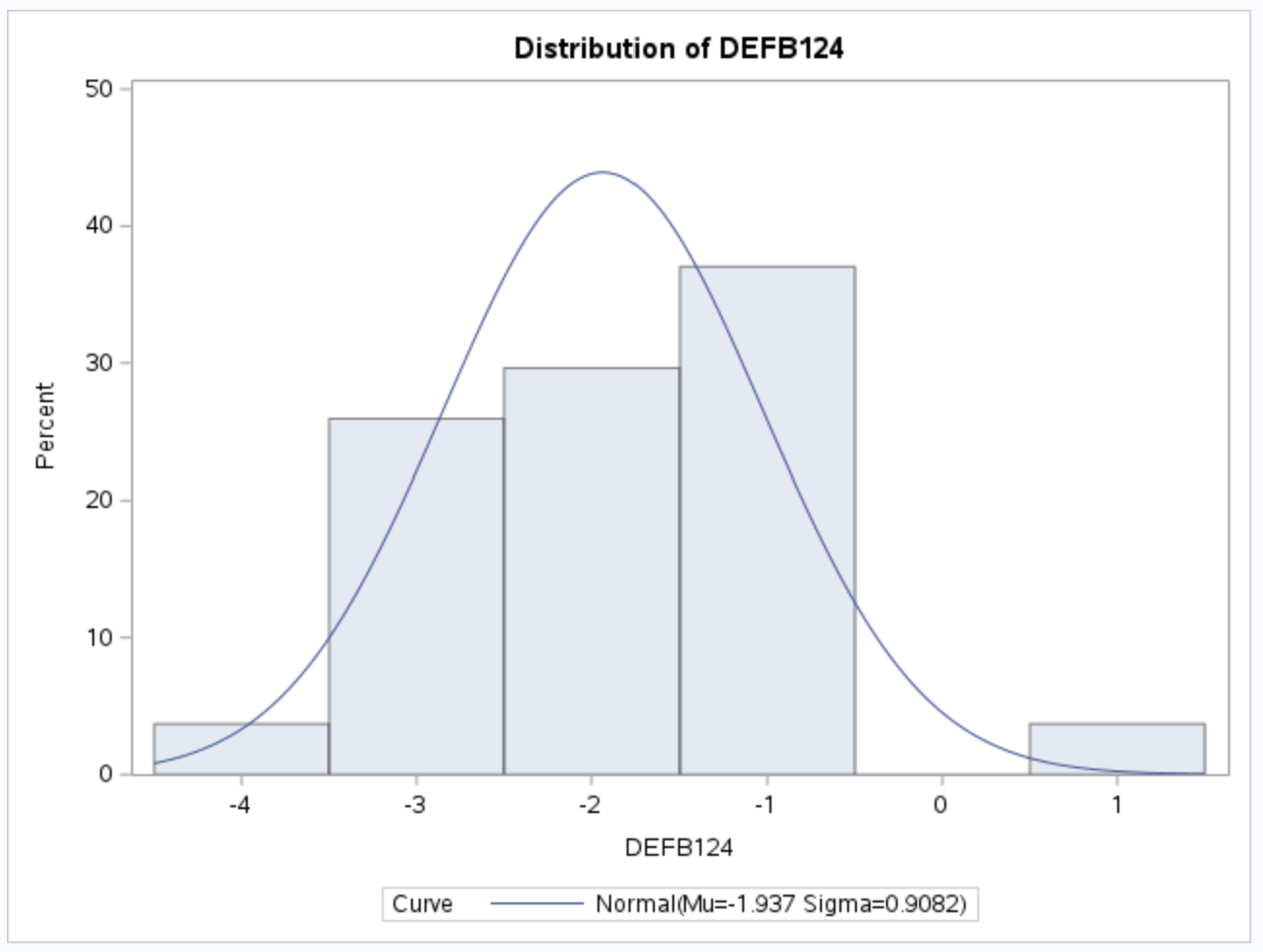
*

*
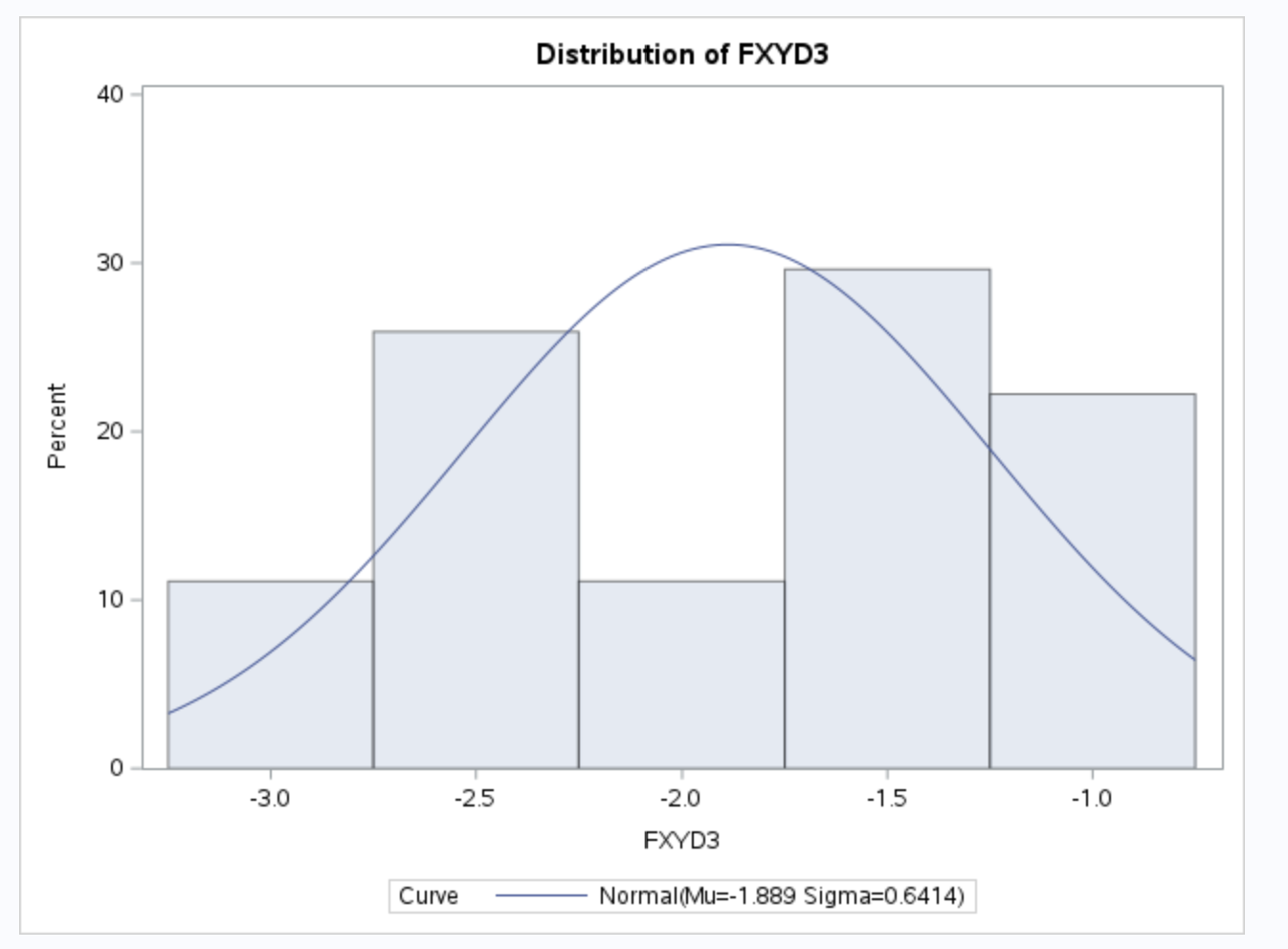
*

|  | Nonparametric P-value With Outliers | Nonparametric P-value Without Outliers |
| --- | --- | --- |
| Total PCB MB vs CB | 0.4397 | 0.4203 |
| Gender wise total PCB in CB | 0.0616 | 0.0977 |
| Gender wise total PCB in MB | 0.9141 | 0.6313 |

*Supplementary Table 13:* *Wilcoxon Rank-Sum (Mann–Whitney U) Test for Total PCB Levels in MB vs. CB and Gender-Wise Differences*

*Supplementary Table 14:* *Wilcoxon Rank-Sum (Mann–Whitney U) Test for Individual PCB Type in MB vs. CB*

| PCB Type | Nonparametric P-value With Outliers | Nonparametric P-value Without Outliers |
| --- | --- | --- |
| PCB202 | 0.03861 | 0.036679 |
| PCB209 | 0.073169 | 0.056815 |
| PCB171 | 0.078867 | 0.07018 |
| PCB172 | 0.145061 | 0.125352 |
| PCB178 | 0.196788 | 0.181704 |
| PCB196 | 0.202236 | 0.204092 |
| PCB206 | 0.225129 | 0.204092 |
| PCB195 | 0.225129 | 0.204092 |
| PCB183 | 0.319599 | 0.298078 |
| PCB199 | 0.334955 | 0.313555 |
| PCB167 | 0.35078 | 0.329552 |
| PCB156 | 0.35078 | 0.329552 |
| PCB170 | 0.392373 | 0.337234 |
| PCB189 | 0.401037 | 0.371813 |
| PCB105 | 0.408062 | 0.380652 |
| PCB194 | 0.409815 | 0.389619 |
| PCB187 | 0.474387 | 0.455908 |
| PCB74 | 0.493813 | 0.465871 |
| PCB177 | 0.493813 | 0.475953 |
| PCB153 | 0.513657 | 0.49647 |
| PCB180 | 0.523732 | 0.506904 |
| PCB138 | 0.565022 | 0.54976 |
| PCB146 | 0.66871 | 0.606429 |
| PCB157 | 0.695739 | 0.679988 |
| PCB118 | 0.767631 | 0.737368 |
| PCB66 | 0.807799 | 0.749315 |
| PCB99 | 0.992413 | 0.927561 |

*Supplementary Table 15:* *Wilcoxon Rank-Sum (Mann–Whitney U) Test for* *Male vs. Female Gene Expression Levels in CB.*

| Genes | Nonparametric P-value With Outliers | Nonparametric P-value Without Outliers |
| --- | --- | --- |
| RPS4Y1 | 1.82E-05 | 2.99E-05 |
| EIF1AY | 3.39E-05 | 6.97E-05 |
| XIST | 0.00077 | 0.000227 |
| S100A8 | 0.005308 | 0.003405 |
| EXOC6B | 0.005705 | 0.010403 |
| PAM | 0.0480 | 0.048098 |

*Figures 11a, 11b and 11c: Relationship of Mother Age and List of Genes that are Significantly Expressed*

11A


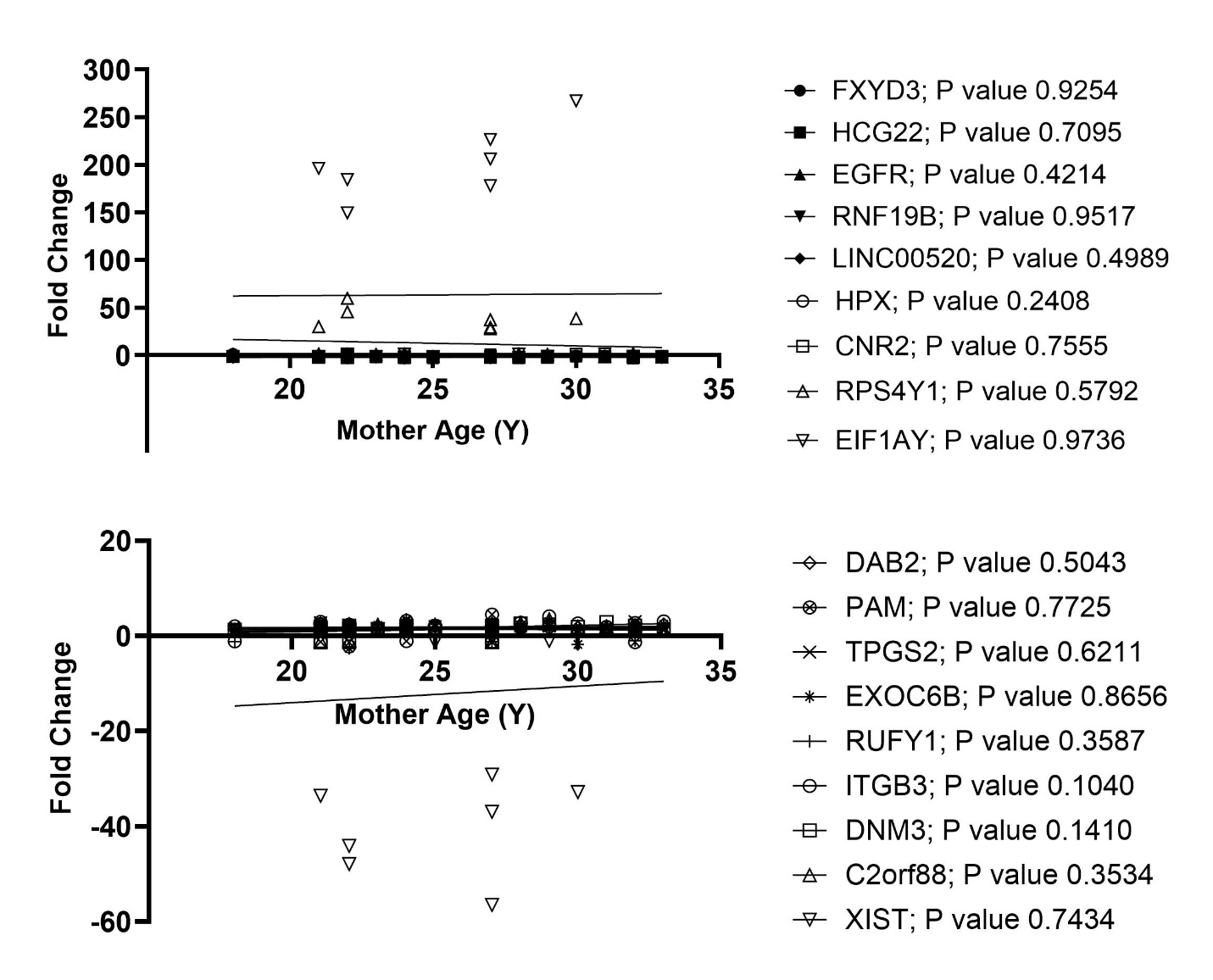


11B


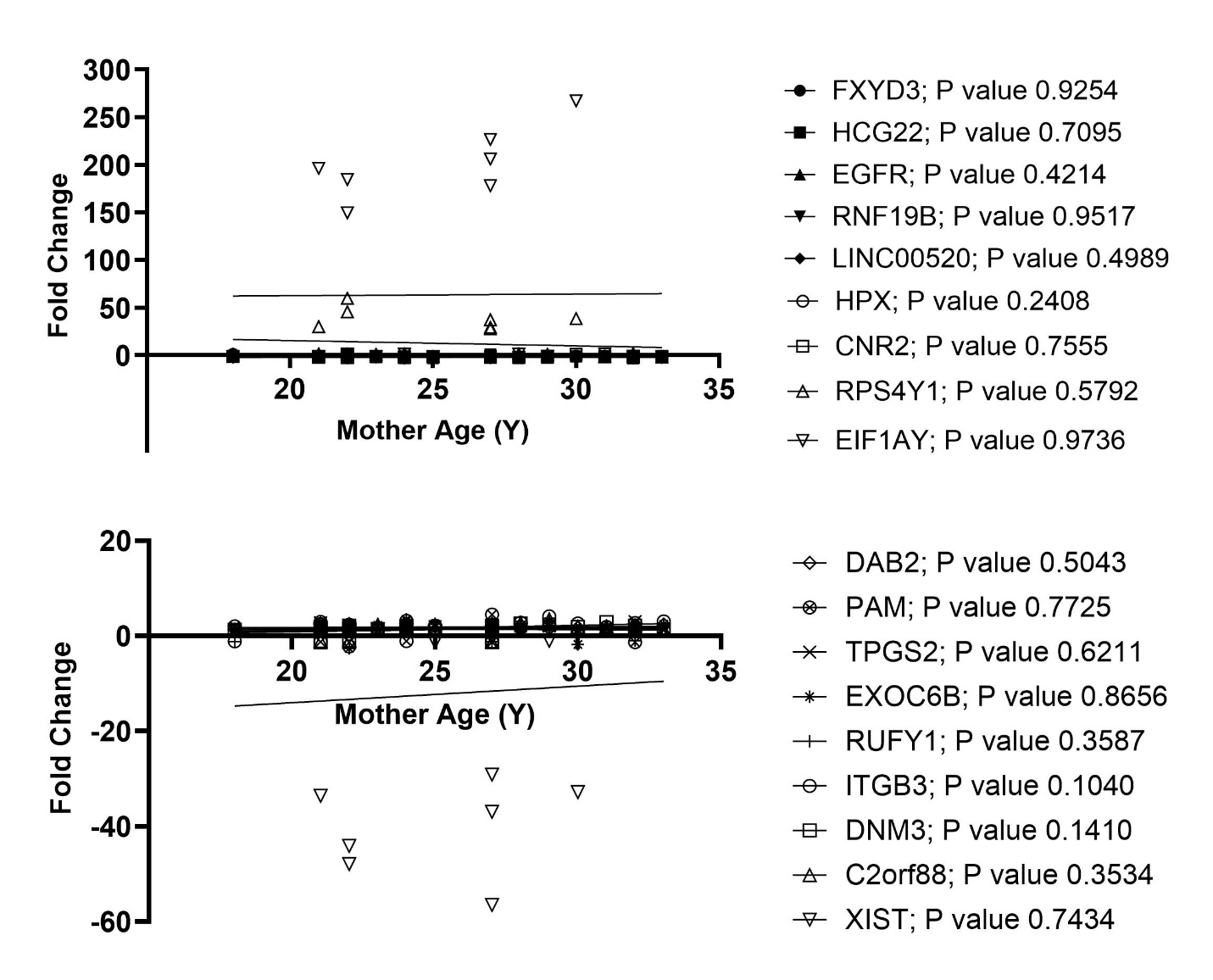


11C


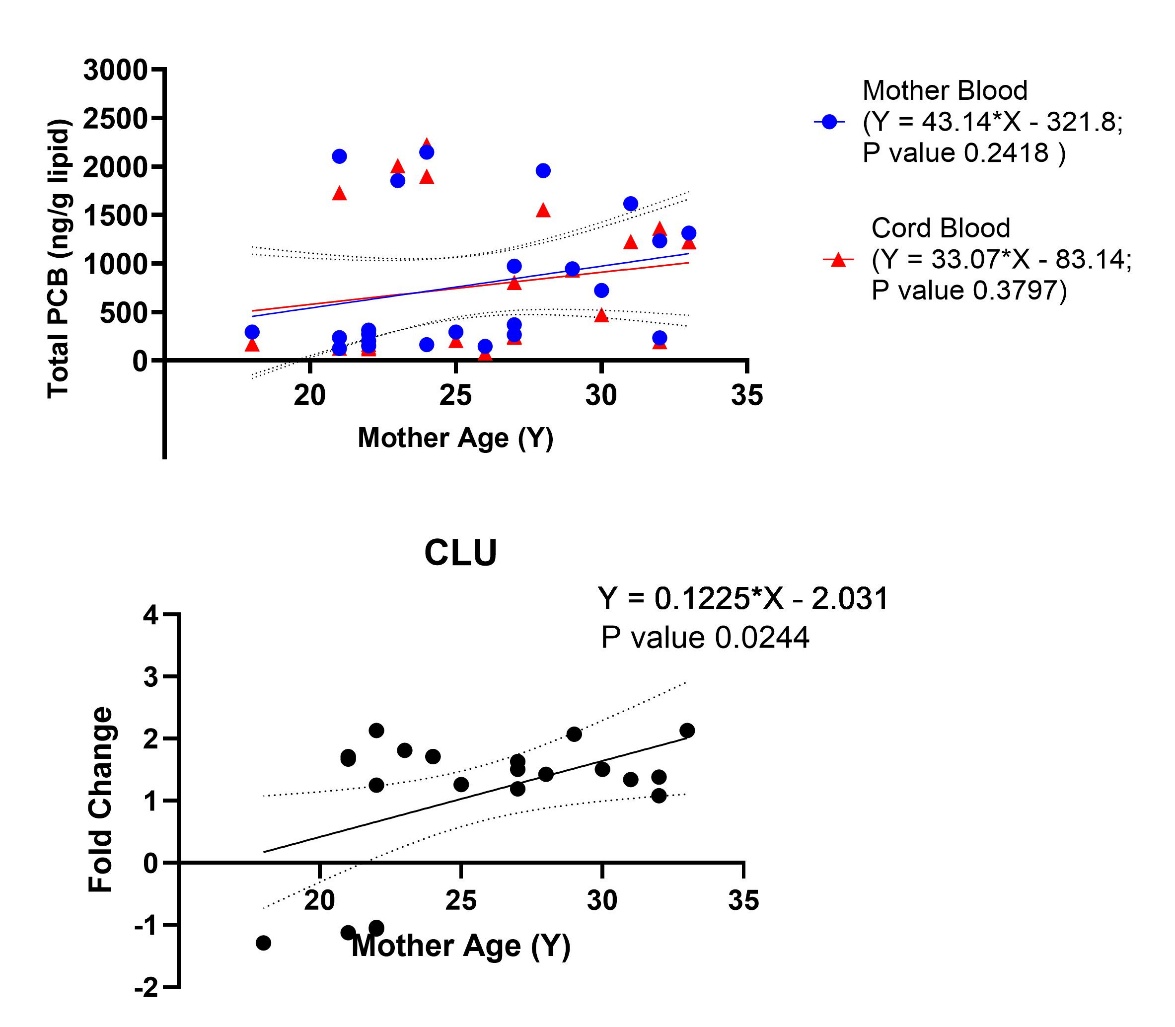


*Figures 12: Relationship of Mother Age and Total PCB in Mother and Cord Blood*


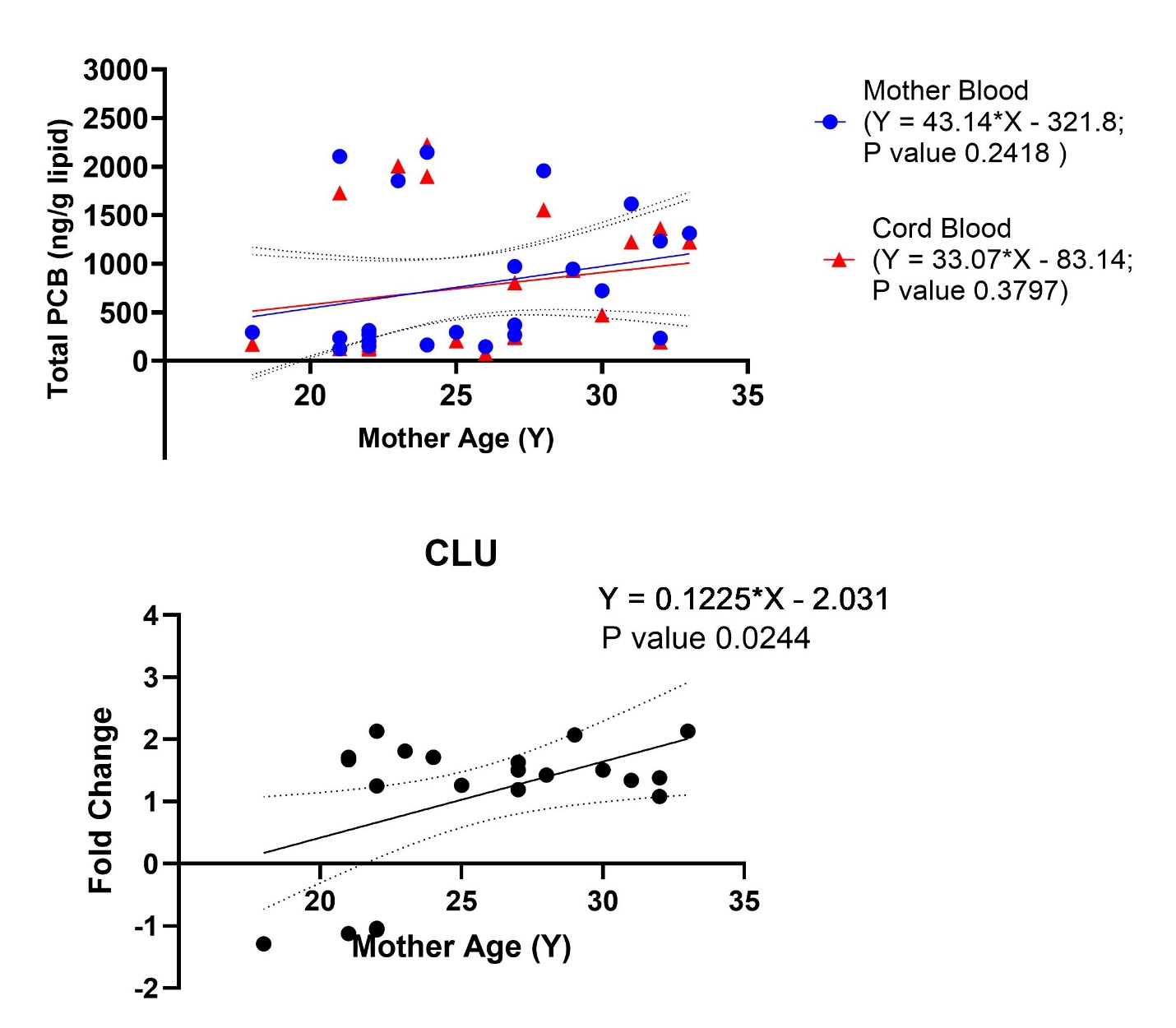

Supplement: 1 [file NIHMS2116983-supplement-1.docx]
